# The buffering capacity of stems: genetic architecture of nonstructural carbohydrates in cultivated Asian rice, *Oryza sativa*

**DOI:** 10.1111/nph.14614

**Published:** 2017-05-30

**Authors:** Diane R. Wang, Rongkui Han, Edward J. Wolfrum, Susan R. McCouch

**Affiliations:** ^1^ Section of Plant Breeding and GeneticsSchool of Integrated Plant SciencesCornell UniversityIthacaNY14853‐1901USA; ^2^ Integrated Biorefinery Research FacilityNational Renewable Energy LabGoldenCO80401USA

**Keywords:** carbon allocation, climate, genome‐wide association, near‐infrared spectroscopy, nonstructural carbohydrates (NSC), *Oryza sativa*, plasticity

## Abstract

- Harnessing stem carbohydrate dynamics in grasses offers an opportunity to help meet future demands for plant‐based food, fiber and fuel production, but requires a greater understanding of the genetic controls that govern the synthesis, interconversion and transport of such energy reserves.
- We map out a blueprint of the genetic architecture of rice (*Oryza sativa*) stem nonstructural carbohydrates (NSC) at two critical developmental time‐points using a subpopulation‐specific genome‐wide association approach on two diverse germplasm panels followed by quantitative trait loci (QTL) mapping in a biparental population.
- Overall, 26 QTL are identified; three are detected in multiple panels and are associated with starch‐at‐maturity, sucrose‐at‐maturity and NSC‐at‐heading. They tag *OsHXK6* (rice hexokinase), *ISA2* (rice isoamylase) and a tandem array of sugar transporters.
- This study provides the foundation for more in‐depth molecular investigation to validate candidate genes underlying rice stem NSC and informs future comparative studies in other agronomically vital grass species.

## Introduction

Synthesis, storage, breakdown and transport of nonstructural carbohydrates (NSCs) constitute a facet of physiology that is fundamental to all plant life. NSCs refer to carbohydrates that are not incorporated into the cell wall and therefore represent a relatively labile internal substrate pool. Due to this inherent plasticity, NSC metabolism plays roles that extend beyond supporting primary plant growth and development; it underlies unique adaptive strategies to long‐ or short‐term environmental shocks. For this reason, there has been particular interest in optimizing stem NSCs in economically important species of the grass family, *Poaceae*, as an approach for varietal improvement in the face of a changing global climate (Slewinski, 2012). Fine‐tuned adjustments to stem NSC levels can lead to a variety of advantageous responses in grass species under abiotic stress. In temperate climates where perennial grasses must overwinter, accumulation of stem carbohydrates serves a protective role against harsh freezing temperatures (Hisano *et al*., 2004). In tropical regions that suffer flash flooding, temporary attenuation of stem NSC metabolism preserves critical energy stores required for regrowth of grasses after waters recede (Singh *et al*., 2014). A contrasting survival strategy is taken in other areas of the tropics where seasonal ‘stagnant flooding’ occurs: deepwater rice cultivars uniquely adapted to this niche environment respond by rapidly consuming their NSC reserves for shoot elongation in order to keep up with steadily rising flood levels (Kato *et al*., 2014). At the opposite end of the environmental spectrum (drought), pre‐anthesis stem NSC has been shown to buffer grain‐filling in both wheat and rice (Yang *et al*., 2001; Saint Pierre *et al*., 2010). Despite the promise of harnessing grass stem reserves to support the development of improved varieties for sustained food, forage and bioenergy production under highly variable environments, progress hangs on a greater understanding of the genetic and regulatory controls that influence the nuances of stem NSC.

In the present study, we focus on cultivated Asian rice, *Oryza sativa*, and elucidate the genetic architecture underlying its stem starch and sucrose contents at two developmental time‐points using a genome‐wide association (GWA) approach. Rice was the first crop genome to be sequenced and has since served as an important model for cereal genetics and as an anchor for comparative studies. In rice, as in most grasses, the net movement of stem NSCs accompanies development. Carbohydrates from photosynthesis un‐utilized for primary sinks (e.g. actively growing organs) are stored in the stem as starch and sucrose before heading; they are remobilized to panicles (inflorescence) during grain‐filling and may be reaccumulated in the stem for a brief period near crop maturity as the panicle loses sink strength. Previous studies investigating the relationship of stem NSC to agronomic traits have linked these carbohydrate reserves to yield potential under optimal conditions (Van Dat & Peterson, 1983) and grain‐filling enhancement (Nagata *et al*., 2002). Additional reports documented the ability of stored stem NSCs to buffer performance under heat and water stress (Yang *et al*., 2001, 2002; Kim *et al*., 2011; Morita & Nakano, 2011). These observations highlight the potential importance of stem NSC for rice yield stability under variable climatic conditions that affect grain‐filling.

Although there is extensive literature on protein‐ and transcript‐level variation that reflect temporal patterns of rice stem NSC (Perez *et al*., 1971; Watanabe *et al*., 1997; Hirose *et al*., 1999, 2006; Ishimaru *et al*., 2004; He *et al*., 2005; Hirano *et al*., 2005; Chen & Wang, 2008; Fu *et al*., 2011; Cook *et al*., 2012), there exists significantly less work describing phenotypic variation observed across accessions (Van Dat & Peterson, 1983; Conocono *et al*., 1995; Samonte *et al*., 2001; Hirano *et al*., 2005; Arai‐Sanoh *et al*., 2011; Pan *et al*., 2011) and even fewer studies that try to disentangle the genetic differences underlying this variation (Nagata *et al*., 2002; Kashiwagi *et al*., 2006; Kanbe *et al*., 2009). Of the genetic studies that have been conducted, all evaluated biparental mapping populations derived from *Japonica* (specifically, the *temperate japonica* subpopulation) × *Indica* (both the *indica* and *aus* subpopulations) crosses, with additional backcrossing to *temperate japonica* as the recurrent parent. Although these studies identified significant quantitative trait loci (QTLs) associated with stem NSC, candidate regions span many megabases and their applicability has been evaluated only in the genetic background of *O. sativa* adapted to temperate rice‐growing regions such as Japan, northern China, Italy, Chile and California (USA). Because the majority of rice production occurs worldwide in the tropics and subtropics, additional investigation is needed to identify QTL associated with NSC levels in tropical rice.

We target our current work on *tropical japonica*, a tropically and subtropically adapted subpopulation of the *Japonica* Varietal Group that is critical to rice production in southern USA, West Africa, South America and parts of Indonesia. We first reveal a coarse understanding of stem NSC genetic architecture by performing a genome‐wide association study (GWAS) on a panel of Southern US rice. This panel represents a specialized gene pool within *tropical japonica* characterized by high linkage disequilibrium (LD) due to shared ancestry from common progenitors. Rice varieties grown in the US are derived from a relatively small number of introductions brought into the country over the course of its < 150‐yr rice production history (Dilday, 1990). We later perform GWAS on a collection of genetically diverse accessions representative of the global *tropical japonica* gene pool using near‐infrared spectroscopy (NIRS)‐predicted phenotypes. This simultaneously provides an opportunity to evaluate QTL discovered in US rice through a higher resolution lens due to reduced LD, and tests the performance of NIR prediction models for rice stem NSC with a previously developed calibration model (Wang *et al*., 2016). Finally, we conduct QTL mapping using an interspecific biparental chromosome segment substitution line (CSSL) library in a *tropical japonica* genetic background (Arbelaez *et al*., 2015). This population comprises near‐isogenic lines (NILs), so that results can lead easily into fine‐mapping efforts. We identify QTL that associate with stem NSC traits in *tropical japonica* rice and highlight promising candidate genes underlying these regions.

Advances in high‐throughput phenotyping methodologies, cost‐effective genotyping assays and accelerated computing power have enabled genetic studies on carbohydrate traits that were not possible in the past. Recently, the first two GWAS studies on biomass constituents in grass species were published. One study focused on bioenergy‐relevant carbohydrate traits in the Andropoganeae model, sorghum (Brenton *et al*., 2016) whereas the other targeted water‐soluble carbohydrates in wheat (Dong *et al*., 2016). The current investigation on NSC in rice adds a complementary dimension to a growing body of knowledge on the genetic control of carbon allocation in grasses.

## Materials and Methods

### Plant materials

Three sets of rice germplasm (*Oryza sativa* L.) were evaluated in this study: US‐TRJ, a US rice panel made up of 126 varieties representative of the *tropical* gene pool of US breeding programs; GLOBAL‐TRJ, a diversity panel composed of 136 *tropical japonica* and 39 admixed *Japonica* accessions from the Rice Diversity Panels 1 and 2 (McCouch *et al*., 2016); and an interspecific CSSL library, which comprised 48 NILs of cv Curinga (subpopulation *tropical japonica*) that each harbored different genomic introgressions from *Oryza rufipogon* accession IRGC105491 (Arbelaez *et al*., 2015). Accession information may be found in Supporting Information Table S1.

### Genotyping information

Genotype data on these panels were generated in previous or ongoing studies. The *tropical japonica* diversity panel had been genotyped for 700 000 single nucleotide polymorphisms (SNP) using the high density rice array (HDRA) (McCouch *et al*., 2016) and the Curinga/*O. rufipogon* CSSL population had been characterized previously using a 6 K SNP array (Arbelaez *et al*., 2015). For US‐TRJ, raw genotyping‐by‐sequencing (GBS) data that were generated as part of a separate, larger study were reanalyzed here to provide SNP data for US‐TRJ genome‐wide association studies (GWAS). The GBS analysis pipeline followed the procedure outlined previously (Spindel *et al*., 2013). Due to the high proportion of missing information inherent in GBS data, we filtered the GBS dataset for 80% call rate and 0.05 minor allele frequency to generate a SNP dataset of 38 618 sites for performing GWAS on the US varieties. Variant information at these 38 618 markers may be found at NCBI's dbSNP (batch ID #1062731; https://www.ncbi.nlm.nih.gov/SNP/snp_viewBatch.cgi?sbid=1062731).

### Evaluation conditions, sample processing and phenotypes

Growth management, destructive sampling and sample processing followed conditions described previously (Wang *et al*., 2016). Briefly, plant stems were collected at two time‐points: heading and physiological maturity. At each sampling point, tillers were excised, leaf blades and panicles removed, and remaining culms microwaved and then slow‐dried over a period of several days until constant weight. The US panel was unreplicated whereas the *tropical japonica* panel and CSSL evaluation measures were averaged across three replicates per accession and two replicates per accession, respectively. All NSC phenotypes were re‐expressed in absolute terms (g) on a per tiller basis. In total, the US panel evaluated four phenotypes (stem starch and stem sucrose at heading and maturity). In the *tropical japonica* panel, six phenotypes were evaluated (starch, sucrose and total nonstructural carbohydrates (TNC) at heading and maturity). In the CSSLs, four phenotypes were evaluated (starch and sucrose at heading and maturity). Phenotype data can be found in Tables S2 and S3 and Methods S1.

### Near‐infrared spectroscopy measurements and carbohydrate assays

NSC components (relative starch and sucrose, % g g^−1^ DW) and TNC (% g g^−1^ DW) were predicted for the 976 samples (485 from heading and 491 from maturity) that were part of the *tropical japonica* diversity panel using NIR spectral data. Calibration models were developed previously on a set of 434 samples of diverse rice stems (Fig. S1); the PLS‐1 model predicted univariate TNC whereas the PLS‐2 model predicted starch and sucrose components at once (Wang *et al*., 2016). The 976 prediction samples were packed in 2‐dram borosilicate scintillation vials and scanned using a Thermo Antaris II FT‐NIR Spectrometer with a 40‐position autosampler carousel (Thermo Fisher Scientific, Waltham, MA, USA). Each sample was scanned 128 times (wavenumber range: 3300–12 000 cm^−1^). For predictions, we used functions found in the following R packages: pls, signal and prospectr. A full description of the prediction analysis, including NIR spectral data and custom R scripts are found within Methods S2.

### Selection of *a priori* candidate genes

One hundred and twenty *a priori* candidate genes were identified based on membership in the following pathways according to RiceCyc (Dharmawardhana *et al*., 2013): sucrose degradation, sucrose synthesis, starch degradation and starch synthesis. All pathways were classified under carbohydrate metabolism > starch and sucrose metabolism. An additional five members of the rice sucrose transporter gene family (OsSUT1‐5) were added to this list, resulting in a total of 125 *a priori* candidate genes (Table S4).

### Statistical analysis

Data handling, analysis and plotting were performed using R statistical software (https://www.R-project.org/). Kinship matrix calculations and genome‐wide association analyses were carried out using functions within the rrBlup R package. For US‐TRJ, we assessed the extent of high‐level population structure using a principal components (PC) analysis (Fig. S2). PC1 explained 21.8% of the total variance, PC2: 5.9%, PC3: 4.8%, PC4: 4.2%. The first PC and kinship matrix were therefore included in the US‐TRJ GWAS model. For GLOBAL‐TRJ association analysis, individuals out of a larger Rice Diversity Panel that shared subpopulation identity were selected to comprise the GLOBAL‐TRJ panel, so that only a kinship matrix was required in this model (McCouch *et al*., 2016). Visualization of results (Manhattan and qq plots) utilized the qqman R package. To deal with multiple testing inherent in GWAS, we set a Benjamini–Hochberg false discovery rate (FDR) threshold of 1% for US‐TRJ and 10% for GLOBAL‐TRJ (Benjamini & Hochberg, 1995). A more stringent FDR was used for the US‐TRJ due to a high frequency of large –log_10_(*P*‐values) diagnosed visually in QQ plots even after controlling for population structure. In the US‐TRJ panel, contrasting with the GLOBAL‐TRJ, swaths of SNPs are perfectly correlated (Fig. S3), leading to plateaus in the QQ plot of SNPs that have the same *P*‐value, in addition to many other SNPs that are high LD and have similar *P*‐values (Fig. S4). Local LD (*r* ^2^) was calculated for the most significant SNP (msSNP) within each association peak resulting from the *tropical japonica* GWAS using Plink1.9 (https://www.cog-genomics.org/plink2; Chang *et al*., 2015). A critical *r* ^2^ was first determined as the 95^th^ percentile of the distribution of all pairwise inter‐chromosomal *r* ^2^ (i.e. unlinked) with the significant SNP marker. Next, pairwise *r* ^2^ values for all markers within 1 Mb of the msSNP were plotted against physical distance from the msSNP. These data were then fitted to a second‐degree Loess curve as described previously (Breseghello & Sorrells, 2006; Laido *et al*., 2014). The intercept of the resultant curve and the critical *r* ^2^ value was determined as the point of local LD decay (Fig. S5). In the *tropical japonica* GWAS, the point of LD decay could not be determined for two of these loci; the msSNPs appeared unlinked to even their closest adjacent SNPs as the Loess curve never intercepted the critical *r* ^2^ value. Candidate regions were further explored using the GWAS Viewer Tool (McCouch *et al*., 2016). Stepwise regression within IciMapping v4.0 software (http://www.isbreeding.net/software/?type=detail&id=14, RSTEP‐LRT option) was implemented to map QTL for stem NSC traits in the interspecific CSSL population on line means (refer to Methods S1 for IciMapping input file). Markers with a logarithm of odds greater than 3.0 were declared significant. For sequence analysis of the *OsIsa2* gene and putative promoter region, sequences on chromosome 5 from 19 154 970 to 19 159 100 bp were extracted for cv Curinga from a previously published resequencing dataset (Duitama *et al*., 2015) and for *O. rufipogon* IRGC105491 from an unpublished resequencing dataset from the McCouch Lab. All sites that were successfully called with respect to the reference genome were aligned and polymorphisms between the two accessions were extracted. The alignments for IRGC105491 are found at NCBI's SRA (short read archives), ID #SRR5440513.

### Analysis of potential functional variants

In order to look for plausible functional variants within putative candidate genes that underlie QTL discovered from GWAS, we extracted insertion/deletion and SNP information for gene sequences plus 1.5 kb upstream of each gene using publically available information from the 3000 Rice Genome Project (‘The 3000 rice genomes project’, 2014) using its 1.0 release (2.3 M indels and 4.8 M SNP datasets).

### Expression information

Transcriptional data for putative candidate genes were extracted from the RiceXPro database (Sato *et al*., 2012b; http://ricexpro.dna.affrc.go.jp/index.html) (Fig. S6).

### Pedigree information

Relationship information on US varieties were derived from Germplasm Resources Information Network (GRIN, http://www.ars-grin.gov/) and Ricebase (Edwards *et al*., 2016). Pedigrees were visualized using Helium (Shaw *et al*., 2014).

### Admixture analysis

Admixture composition analyses of select individuals harboring the minor allele for QTL peak markers were performed using RFMix (Maples *et al*., 2013) after imputation by fastPHASE (Scheet & Stephens, 2006). Because rice is inbred and nearly 100% homozygous, genotypes were used directly as haplotypes. Data formatting for input files for these software were facilitated by the use of custom Perl scripts released previously (Brenton *et al*., 2016).

## Results

### Stem NSC association analysis in US rice

To gain a rough understanding of rice stem NSC genetic architecture, a collection of 126 *tropical japonica* accessions (hereafter referred to as US‐TRJ) and seven *temperate japonica* varieties that represent the historical genetic diversity of US rice production (Table S1) were phenotyped for four NSC component traits (starch and sucrose at heading and maturity). In US rice breeding programs, crossing occurs primarily within market classes that are defined by grain type, that is, long, medium or short grain. This practice is reflected in a partitioning of genetic variation by market class (Lu *et al*., 2005). To avoid confounding phenotype–genotype associations with genetic population structure, we first checked to see if stem NSC phenotype distributions within US‐TRJ were significantly correlated with the two market classes represented in the US‐TRJ gene pool, namely long and medium grain types (note: short grain varieties in this study belong to the *temperate japonica* subpopulation*,* grown almost exclusively in California). There was no observed alignment of NSC phenotypes with long and medium grain market classes (Fig. S7). We proceeded with association mapping in US‐TRJ for the four NSC traits but included population stratification controls and a stringent FDR threshold (FDR = 0.01) as precautions against Type 1 error.

**Figure 1:**
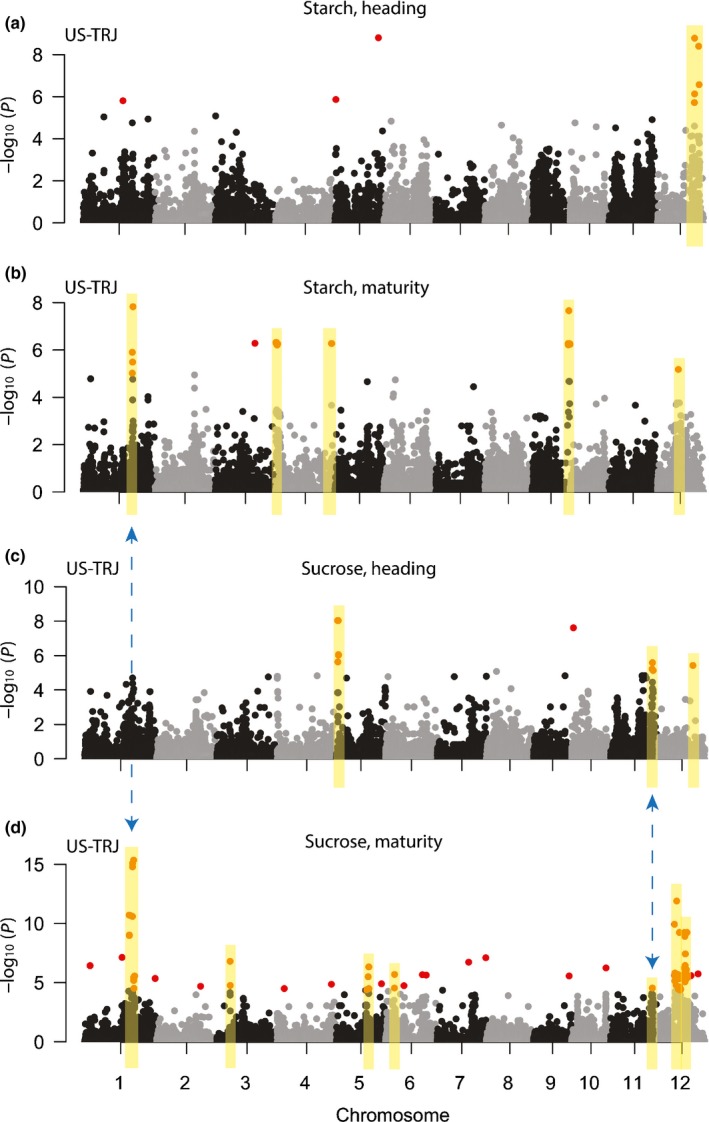
Genetic architecture of stem nonstructural carbohydrates (NSC) in US *tropical japonica* rice. Genome‐wide association results for stem NSC traits in US‐TRJ (126 *tropical japonica* varieties): (a) starch at heading, (b) starch at maturity, (c) sucrose at heading and (d) sucrose at maturity. Red circles, single nucleotide polymorphisms (SNPs) significant at 0.01 false discovery rate (FDR); yellow boxes, peaks supported by at least two significant SNPs that localize within 500 kb of each other; blue dashed arrow, regions on chromosomes 1 and 11 that are significant across traits. Note that some regions highlighted in yellow appear to be singleton SNPs but are actually multiple, closely linked markers that overlap visually (Supporting Information Table S19).

From US‐TRJ results, we cataloged 15 regions of association, defined by support from multiple significant SNPs within 500 kb of each other (Figs 1, S3; Table S5). There were two loci associated with starch‐at‐heading on chromosome 12, four associated with starch‐at‐maturity on chromosomes 1, 4, 9 and 12, three associated with sucrose‐at‐heading on chromosomes 5, 11 and 12, and six associated with sucrose‐at‐maturity on chromosomes 1, 3, 5, 6, 11 and 12. In addition, we checked to see if remaining singleton significant SNPs co‐localized within 500 kb of significant regions of other NSC traits, but they did not. To test for the effect of phenology on stem NSC genetic architecture, we repeated US‐TRJ GWA analyses with the addition of a days‐to‐heading (DTH) covariate. For starch‐at‐maturity, sucrose‐at‐heading and sucrose‐at‐maturity, resultant GWAS *P*‐values corresponded well with analyses that did not account for DTH (*r *=* *0.90–0.99). For starch‐at‐heading (*r *=* *0.77), most of the genetic signal was muted by the addition of the DTH covariate; however, one QTL on chromosome 12 remained significant at 0.05 FDR (Fig. S8).

Of the 15 cataloged US‐TRJ QTL for stem NSC, two regions appeared to co‐localize across multiple traits. The first was around the midpoint of chromosome 1 where a significant locus was associated with both sucrose and starch at maturity. The msSNP for sucrose‐at‐maturity (S1_29495819) was found at position 29 495 819 bp and the msSNP for starch‐at‐maturity (S1_29976759) was 500 kb downstream at position 29 976 759 bp. Despite the large physical distance between these two msSNPs, it is possible that they may be tracking the same causative factor due to high LD in US rice (Fig. S9). The second significant region that co‐localized across traits was found on chromosome 11 at the 25‐Mb position. Sucrose‐ and starch‐at‐maturity were associated with this locus and both traits shared exactly the same msSNP. The remaining 11 QTL found in US‐TRJ were trait‐specific. Although performing GWAS using high LD populations such as US‐TRJ requires lower marker density for QTL discovery, the trade‐off is low mapping resolution, so we could not acceptably make further inferences without additional evaluations.

### NSC prediction of GLOBAL‐TRJ using NIR spectral information

**Figure 2:**
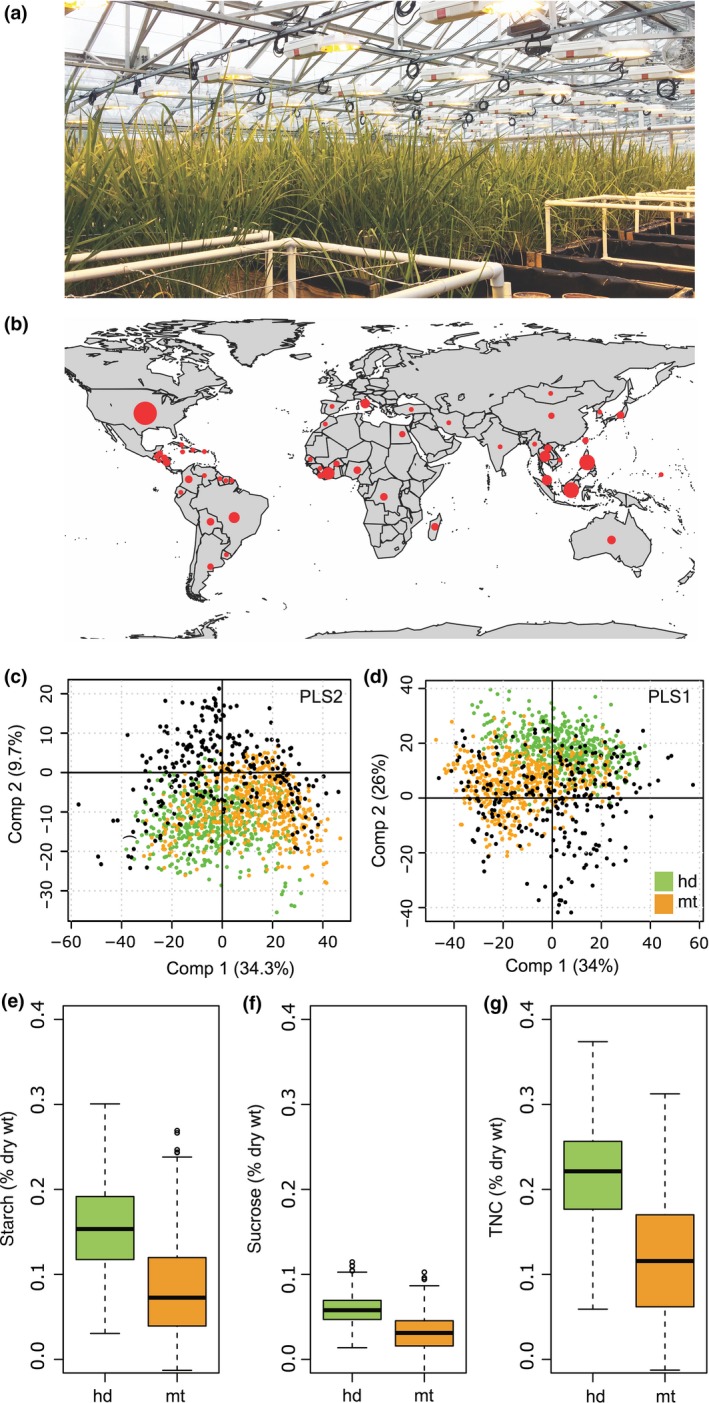
Evaluation of global *tropical japonica* rice accessions for stem nonstructural carbohydrates (NSCs) using predictions by near‐infrared spectral information. (a) Photo of GLOBAL‐TRJ panel growing in glasshouse. (b) Map showing geographical origin of the 175 GLOBAL‐TRJ samples. (c) Distribution of 976 prediction samples (green and orange circles) and 300 calibration samples (black circles) in PC1 and PC2 of partial least square (PLS) model space using spectral data for PLS2 and (d) PLS1 models. Distribution of predicted values, expressed in percentage dry weight (DW), at heading (hd) and maturity (mt) for (e) starch, (f) sucrose and (g) total nonstructural carbohydrates (TNC). Edges of boxplots found in (e–g) are the first and third quartiles of the distributions, bars represent the highest and lowest value within the 1.5 interquartile range from the first and third quartiles, and open circles represent observations beyond those values.

In order to gain a finer understanding of the genetic architecture underlying stem NSC in *tropical japonica* rice and to evaluate whether the 15 QTL discovered in US rice were significant beyond these regional breeding programs, we phenotyped a geographically and genetically diverse *tropical japonica* panel of 175 accessions, hereafter referred to as GLOBAL‐TRJ (Fig. 2a,b; Table S1). This collection of diverse landraces has much lower LD than US‐TRJ (Fig. S3), thereby supporting greater mapping resolution. In lieu of wet chemistry assays, we scanned the set of *tropical japonica* stem samples for NIR reflectance and made predictions of NSC components using the resultant spectral data as a higher‐throughput approach to determine stem constituents (Methods S2). Starch and sucrose levels (% g g^−1^ DW) were jointly predicted using a single PLS‐2 model whereas TNC (% g g^−1^ DW), were predicted separately using a PLS‐1 model. We found that spectral data measured on our prediction set were well‐aligned to that of the calibration set (Figs 2c and S1), and so we were able to predict the three NSC constituents across two developmental points with acceptably low uncertainty estimates. Predicted values at heading and maturity were consistent with expected distributions (i.e. higher means at heading than maturity) (Fig. 2d), and were adjusted for stem DW to yield absolute estimates of NSC per tiller for subsequent GWA analyses.

### Association analysis of *tropical japonica* rice stem NSC

Out of 700 000 genome‐wide SNP markers found on the HDRA (McCouch *et al*., 2016), 222 689 had minor allele frequencies >0.05 in GLOBAL‐TRJ and were used in conjunction with six NIR‐predicted traits (starch, sucrose, and TNC at heading and maturity) for GWAS. Heritability estimates (Table S6) for these traits in rice were lower than previously reported (Wang *et al*., 2016), likely due to glasshouse anomalies observed during this evaluation. These genome scans yielded a total of nine loci of interest (Fig. S10; Table S7): three were associated with starch‐at‐heading, two with starch‐at‐maturity, two with TNC‐at‐heading, one with TNC‐at‐maturity and one QTL on chromosome 1 associated with both starch‐at‐maturity and sucrose‐at‐maturity. Local LD decay was calculated for each of these regions (see Methods; Table S7), resulting in values that ranged from 33.85 to 252.78 kb on either side of the msSNP (corresponding to between six and 50 gene models not annotated as transposable elements. Of these nine loci, two appeared to co‐localize with peaks resulting from US GWA analyses and warranted further investigation; they were found on chromosomes 1 and 11 (Table 1) and additionally corresponded to the same loci found to co‐localize across traits within US‐TRJ.

**Table 1:** Genome‐wide association peaks common across US‐TRJ and GLOBAL‐TRJ panels of *Oryza sativa*

Trait (GLOBAL‐TRJ)	Marker (GLOBAL‐TRJ)	Chr	Marker position (bp)	Local LD (kb)	Candidate region size (kb)	No. of gene models (non‐TE)	Candidate gene(s)
Starch, mt	SNP‐1.30962744	1	30 963 789	48.99	97.98	13	Hexokinase (OsHXK6)
TNC, hd	SNP‐11.24673979	11	25 140 140	252.87	505.74	50	4 PLT sugar transporters

LD, linkage disequilibrium; TE, transposable element; TNC, total nonstructural carbohydrates; mt, maturity; hd, heading.

**Figure 3:**
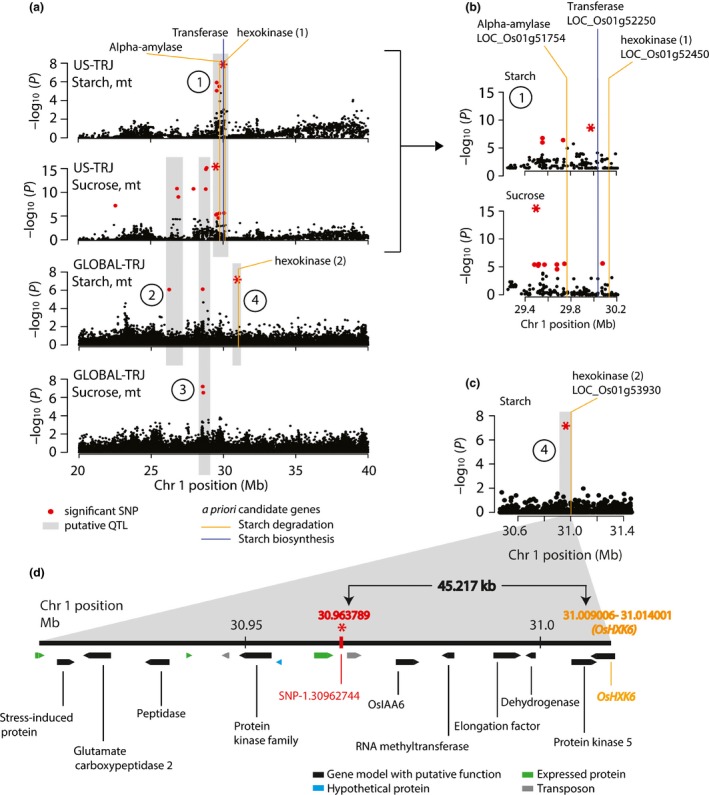
Dissection of a region on chromosome 1 associated with stem nonstructural carbohydrates (NSC) at maturity in rice, *Oryza sativa*. (a) Zoomed‐in view of target region (chr1: 20–40 Mb; Fig. 1 and Supporting Information Fig. S6) with four distinct subquantitative trait loci (QTL) (gray boxes labeled 1–4, circled). QTL‐1 is significantly associated with starch and sucrose in US‐TRJ but not in GLOBAL‐TRJ. QTL‐2 is shared between sucrose in US‐TRJ and starch in GLOBAL‐TRJ. QTL‐3 is significantly associated with sucrose in US‐TRJ and both starch and sucrose in GLOBAL‐TRJ. QTL‐4 is significant only in starch‐at‐maturity in GLOBAL‐TRJ. Vertical lines mark the locations of *a priori* candidate genes and line colors indicate the candidate genes’ biochemical pathway (orange, starch degradation; blue, starch biosynthesis genes). (b) Zoomed‐in view of the 1‐Mb region containing QTL‐1 defined by most significant single nucleotide polymorphism (msSNP) ‘S1_29976759’ for starch and msSNP ‘S1_29495819’ for sucrose (red asterisks). Three *a priori* genes (alpha‐amylase = LOC_Os01g51754; transferase = LOC_Os01g52250; hexokinase(1) = LOC_Os01g52450) summarized in Table S5. (c) Zoomed‐in view of 1‐Mb region surrounding msSNP ‘SNP‐1.30962744’ (red asterisk) in QTL‐4; gray box delimits region of local linkage disequilibrium (LD) (97.98 kb) with the msSNP. (d) Close‐up of the LD region of QTL‐4. 13 nontransposable element (TE) gene models (Table S9) were found here (black, blue and green, non‐TE gene models; gray, TEs). *A priori* candidate gene model *OsHXK6* (LOC_Os01 g53930) labeled in orange; msSNP ‘SNP‐1.30962744’ (red asterisk). Distance between msSNP and start position of *OsHXK6* is 45.217 kb.

Upon closer study, the region on chromosome 1 at *c*. 29–30 Mb, which was associated with starch‐at‐maturity and sucrose‐at‐maturity in both the GLOBAL‐TRJ and US‐TRJ panels, appeared to be a cluster of at least four distinct sub‐QTL that did not co‐localize in the same LD blocks (Fig. S9). We tagged the four putative subregions of significance (QTLs 1–4; Fig. 3a) and inspected them individually. QTL‐1 was found only in US‐TRJ and spanned a *c*. 500‐kb physical interval (Fig. 3b), consistent with the high LD in US rice (Fig. S3). QTLs ‐2 and ‐3 were found in both the US‐TRJ and GLOBAL‐TRJ panels, and QTL‐4 was found in the GLOBAL‐TRJ alone (Fig. 3a). In all cases, the local LD blocks were distinct and nonoverlapping, supporting the idea that this mega‐locus on chromosome 1 is made up of several distinct QTL. For the three QTL detected in GLOBAL‐TRJ, we were able to investigate at a finer scale due to reduced LD, and it was of interest to note that for the msSNPs anchoring QTLs 2–4 in GLOBAL‐TRJ (SNP‐1.26243779, SNP‐1.28557570 and SNP‐1.30962744), the minor allele in the population was consistently associated with increased stem NSC (Fig. S10).

Using the HDRA SNPs that were polymorphic at a minor allele frequency above 5% in GLOBAL‐TRJ, we estimated local LD decay around the msSNPs of QTLs 3 and 4 (Table S7). QTL‐2 was one of the few SNPs for which we could not estimate local LD using our method, potentially due to low regional marker density. The LD regions of QTL‐3 and QTL‐4 encompassed 24 and 13 non‐TE gene models, respectively. No genes with obvious carbohydrate functions were found within the region of LD for QTL 3, but QTL 4 encompassed one *a priori* candidate gene, a hexokinase gene (*OsHXK6*; LOC_Os01g53930) (Fig. 3c). Plant hexokinases function primarily to catalyze the phosphorylation of alpha‐D‐glucose that results from starch breakdown, but perhaps more interestingly, they also have been shown to mediate sugar signaling by binding glucose along with protein factors to repress gene transcription (Jang *et al*., 1997; Granot, 2008; Cho *et al*., 2009). In rice there are 10 hexokinase genes and *OsHXK6* is one of the two documented to play a sugar‐sensing role. Specifically, OsHXK6 was shown to repress transcription of rice alpha‐amylase genes, contributing to a negative feedback under high glucose levels (Cho *et al*., 2009). Using an independent panel of diverse *tropical japonica* from a published resequencing project, we looked for SNPs and Indels within *OsHXK6* and its 1.5‐kb upstream region to catalog potential functional polymorphisms (The 3000 Rice Genomes Project, 2014). We discovered one nonsynonymous SNP and one 3‐bp indel in the coding region and five SNPs in the putative promoter (Table S8). In addition to *OsHXK6*, the LD region of QTL‐4 also included 12 other non‐TE gene models (Fig. 3d; Table S9); none of them had obvious roles related to carbohydrate metabolism, leaving *OsHXK6* as the strongest candidate.

**Figure 4:**
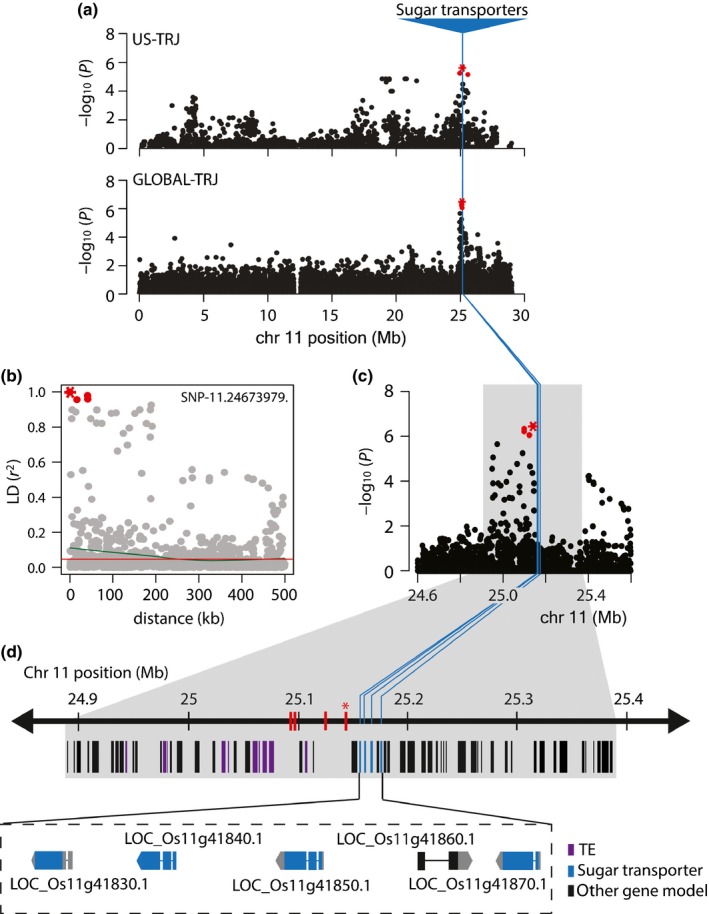
Four sugar transporters underlie a significant peak for nonstructural carbohydrates (NSCs) at heading in rice, *Oryza sativa*. (a) Association analysis revealed a significant peak on chromosome 11 at position 25 Mb in both US‐TRJ for sucrose at heading and in GLOBAL‐TRJ for total NSCs at heading. Significant single nucleotide polymorphisms (SNPs) colored red. (b) Plot showing linkage disequilibrium (LD) decay from the most significant SNP (msSNP) in GLOBAL‐TRJ. Red line indicates the critical *r* ^2^ threshold; green curve shows local decay *c*. 252 kb from msSNP ‘SNP‐11.24673979’ in GLOBAL‐TRJ (Supporting Information Table S7). (c) Zoomed‐in view of 1‐Mb candidate region. Gray box, 505.74‐kb region of LD flanking msSNP ‘SNP‐11.24673979’; blue lines, locations of four tandemly arrayed sugar transporters (LOC_Os11g41830.1, LOC_Os11g41840.1, LOC_Os11g41850.1 and LOC_Os11g41870.1) (Johnson & Thomas, 2007). (d) Thirty‐eight gene models, which are not transposable elements (Table S10). Red lines denote significant SNPs; msSNP ‘SNP‐11.24673979’ (red asterisks). Note that this region was also significant for US‐TRJ sucrose‐at‐maturity (Fig. 1d); zoomed‐in view is not shown here because its msSNP is identical to US‐TRJ sucrose‐at‐heading.

We next investigated the QTL on chromosome 11 at the 25‐Mb position (Figs 4a,c, S11). This region was significant for sucrose in the US‐TRJ and TNC in the GLOBAL‐TRJ. The msSNP here (SNP‐11.24673979) was in a region of local LD that spanned 252.87 kb to each side in GLOBAL‐TRJ (Fig. 4b), and the minor allele was associated with increased stem NSC (Fig. S12b). The region of LD (chr 11: 24 997 270–25 393 010 bp) harbored 12 TEs and 52 non‐TE gene models (Table S10). Though none were genes collected in our *a priori* candidate gene search (Table S4), there were four tandemly arrayed sugar transporters (LOC_Os11g41830, LOC_Os11g41840, LOC_Os11g41850 and LOC_Os11g41870) (Fig. 4d). These genes are part of the polyol/monosaccharide transporter (PLT/PMT) family of monosaccharide sugar transporters (MSTs) in rice (Johnson & Thomas, 2007). MST families are ancient gene families that have expanded and diverged both across and within species due to duplication events; this has given rise to a wide range of functional variation including phloem transport and plant defense (Johnson & Thomas, 2007; Slewinski, 2011). In the rice genome, there are six PLT genes, four of which are tandemly arrayed on chromosome 11. All four underlie the significant region associated with NSC at heading in US‐TRJ and GLOBAL‐TRJ rice identified in this study, although only three are annotated as sugar transporters in the MSUv7 assembly (LOC_Os11g41830 was not). When we cross‐referenced with a second assembly, RAP‐DB (http://rapdb.dna.affrc.go.jp/index.html), we found that LOC_Os11g41830 (Os ID: Os11g0637000) was annotated as a sugar transporter protein. The molecular functions of these PLT transporters have yet to be characterized but based on the results of this study, it is of interest to elucidate whether their roles and regulation have diverged in the context of rice stem carbohydrate accumulation. Of the four tandemly arrayed MSTs, LOC_Os11g41850 and LOC_Os11g41870 were the most conserved in *tropical japonica* (Tables S11–S14). Out of the 54 gene models localized in this region, the three PLT sugar transporters are the most promising candidates in relation to rice stem NSC levels.

In order to better understand the distribution of the rare alleles that were associated with increased NSC levels in *tropical japonica* germplasm, we examined the allele frequencies of SNP‐1.30962774 and SNP‐11.24673979 across a wider spectrum of *O. sativa* diversity*,* beyond *tropical japonica*. Analysis of allele frequencies across 1493 diverse *O. sativa* accessions from the Rice Diversity Panels 1 and 2 (McCouch *et al*., 2016) confirmed that the alleles associated with increased NSC at both of the msSNP loci occurred at low frequencies in *tropical japonica* (7% of *n *=* *279 and 10% of *n *=* *340 for SNP‐1.30962774 and SNP‐11.24673979, respectively). The NSC‐increasing allele for SNP‐1.30962774 was found at higher frequencies in the *indica* and *aromatic* subpopulations (29% and 36%), and the trait‐increasing allele of SNP‐11.24673979 was found at highest frequency in *indica* and *aus* (19.5% and 26%) (Table S15). However, no introgressions from these subpopulations were detected using admixture analysis for either QTL. Examining cumulative effects of QTL on chromosomes 1 and 11 was precluded by small sample size for genotypes that harbor minor alleles at both loci; however, we noted that lines carrying rare alleles at both QTL appear to have a higher level of stem NSC across all traits compared with lines carrying other combinations (Fig. S13).

### QTL mapping in Curinga × wild CSSL population

**Figure 5:**
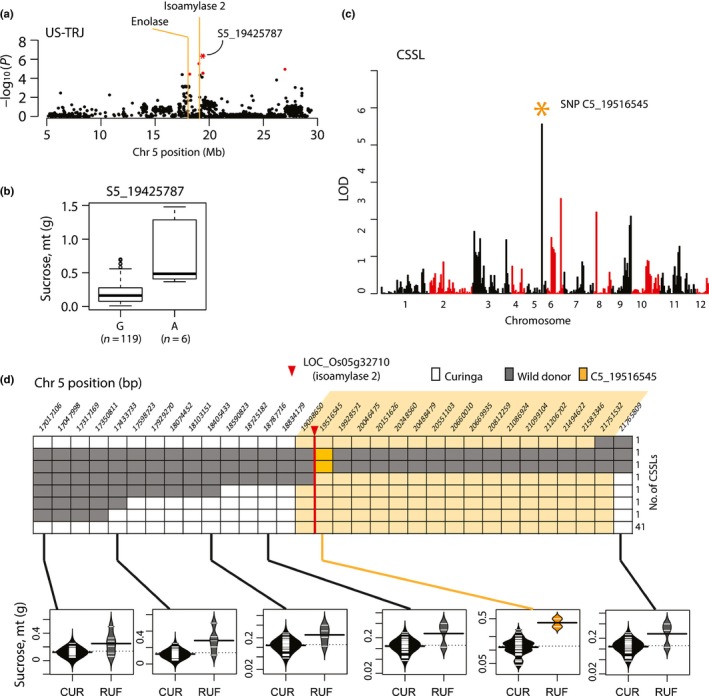
Quantitative trait loci (QTL) on chromosome five is associated with stem sucrose levels at maturity in rice, *Oryza sativa*. (a) Zoomed‐in view showing genome‐wide association study (GWAS) peak on chromosome 5 in US‐TRJ sucrose‐at‐maturity. Red circles, significant single nucleotide polymorphisms (SNPs); orange lines, *a priori* candidate genes, enolase (LOC_Os05g31110) and isoamylase‐2 (LOC_Os05g32710) (Supporting Information Table S4). (b) Boxplot showing sucrose‐at‐maturity distributions for lines carrying the G (*n *=* *119) or A (*n *=* *6) allele at the most significant SNP (msSNP) ‘S5_19425787’. (c) Mapping results from the chromosome segment substitution line (CSSL) population for sucrose‐at‐maturity showing significant marker (c5_19516545, orange asterisk) on chromosome 5 at 19.5 Mb. (d) Graphical genotype showing introgressions in CSSLs in the region around SNP S5_19425787 using data from a 6 K SNP Infinium array. Sucrose distributions (g) are shown below for individuals carrying recurrent parent genotype ‘CUR’, and donor parent genotype ‘RUFI’, at six SNP markers across the region. Bright orange, peak SNP (C5_19516545); pale orange, candidate region associated with phenotype; red arrow, location of *a priori* candidate gene, *OsISA2*.

We next performed QTL mapping in a CSSL population derived from an interspecific cross between cv Curinga, an upland *tropical japonica* variety from Brazil that served as the recurrent parent, and a wild *aus*‐like *O. rufipogon* donor accession (Arbelaez *et al*., 2015; Kim *et al*., 2016). Here we were interested to determine whether QTL associated with stem NSC traits in the CSSLs co‐localized with any regions identified from GWAS in the US‐TRJ and/or GLOBAL‐TRJ. Two QTLs were identified in the CSSLs on chromosome 5 (Table S16); one at 19.5 Mb that was associated with sucrose‐at‐maturity and one at 21.7 Mb that was associated with sucrose‐at‐heading. Of these two CSSL QTLs, the one at 19.5 Mb was found to co‐localize with a GWAS peak in US‐TRJ that was also associated with sucrose‐at‐maturity. We prioritized this region for further investigation. Despite low numbers of individuals carrying the donor genotype relative to the recurrent parent genotype at any particular locus in the CSSL population, we documented a significant effect on stem sucrose‐at‐maturity for a large introgression at the 19.1–21.75‐Mb position. In this case, the *O. rufipogon* allele significantly increased the NSC phenotype. In other words, CSSL lines that harbored a wild *aus*‐like introgression across the target region had values beyond (greater than) the rest of the CSSL lines that carried the *tropical japonica* genotype (Fig. 5c,d). This introgression contained a large number of genes (61 TEs and 339 non‐TEs) so further fine mapping is required to narrow down the target region and determine the causative gene(s). To lay the foundation for this work, line CURUF25, one of the two CSSLs that harbored the donor allele across the region of interest, was backcrossed again to Curinga. We confirmed the genotype of a single F_1_ plant using 1747 polymorphic markers from a 6 K SNP array (Table S17) and generated F_2_ seed for future mapping experiments.

The US‐TRJ GWAS peak that centered at 19.5 Mb on chromosome 5 mapped within the introgressed region of interest in the CSSLs, and contained an *a priori* gene, LOC_Os05g32710 (Fig. 5a,b). The other 339 gene models in the introgressed region had no obvious carbohydrate metabolism roles so we focused our attention on LOC_Os05g32710. Based on our initial *a priori* candidate screen using RiceCyc (Dharmawardhana *et al*., 2013) and the MSUv7 genome assembly (http://rice.plantbiology.msu.edu/), this gene was initially predicted to encode an alpha amylase. However, after investigating previously published experimental work involving this gene, we found that LOC_Os05g32710 is a rice homolog of Arabidopsis *isa2*; it encodes an isoamylase‐2 de‐branching enzyme, ISA2 (Kharabian‐Masouleh *et al*., 2011; Streb & Zeeman, 2014). We checked two additional databases, RiceFREND (Sato *et al*., 2012a) and OryzaCyc (http://www.plantcyc.org/databases/oryzacyc/4.0), and noted that both of these resources annotated LOC_Os05g32710 correctly as an isoamylase. Isoamylases are debranching enzyme complexes that function in the starch biosynthesis pathway to mediate the production of amylopectin, a branched starch molecule (Tetlow *et al*., 2004).

We compared sequences across a *c*. 3‐kb region that included the *ISA2* gene and a 1.5‐kb putative promoter region in the CSSL parents, Curinga and *O. rufipogon*. We found one SNP in the coding sequence and three single base‐pair indels in the putative promoter (Fig. S14). Interestingly, we also found a region in the putative promoter just upstream of the *ISA2* start site that was rich in consecutive missing data points in Curinga. To clarify whether this result was specific to Curinga, or was a more general feature of *tropical japonica* genomes, we examined resequencing information from 484 other *tropical japonica* accessions from the 3000 Rice Genomes Project and discovered that the same region was characterized as a 22‐bp indel that was rich in consecutive Gs (Table S18; Fig. S14). Viewing this region in the MSUv7 genome browser (http://rice.plantbiology.msu.edu/cgi-bin/gbrowse/rice/), we identified a previously annotated (CGGGGG)n repeat (Fig. S14), adding to our confidence that there is a repetitive element in the *ISA2* promoter. These results raise the possibility that there may be transcriptional variation for *ISA2* in *tropical japonica* rice due to the presence of repeat motif differences in the promoter of the *ISA2* gene.

## Discussion

Despite long‐established ideas of tailoring carbon allocation dynamics in agricultural grass species to support production under variable climates, we are still in the early stages of understanding the natural genetic variation responsible for inter‐ and intraspecific phenotypic differences in stem NSC. Consequently, our primary objectives here were to determine the genetic architecture of rice stem NSC traits at the genome level and provide a foundation for investigating hypotheses at the gene level. We demonstrate that GWA is a suitable method to capture stem NSC QTL underlying within‐subpopulation variation in rice and identify three promising genomic regions for NSC traits that co‐localize across germplasm panels. To date, this is the most comprehensive genetic study on rice stem NSC with respect to germplasm evaluated, genetic resources leveraged, and analytical methods employed. It provides a link between past classical physiology experiments that have rigorously examined enzyme and transcript activities associated with rice carbon dynamics, and the wealth of current and forthcoming high‐throughput information. As public data repositories become flush with ‐omics data, marker coordinates and significance scores resulting from these GWA analyses (Tables S19, S20) can be integrated with candidate gene information embedded in new, heterogeneous ‘big datasets’ and reanalyzed in novel ways to construct metabolic networks underlying rice stem NSC, prioritize candidate gene validation, and open the door to more targeted gene discovery efforts.

**Figure 6:**
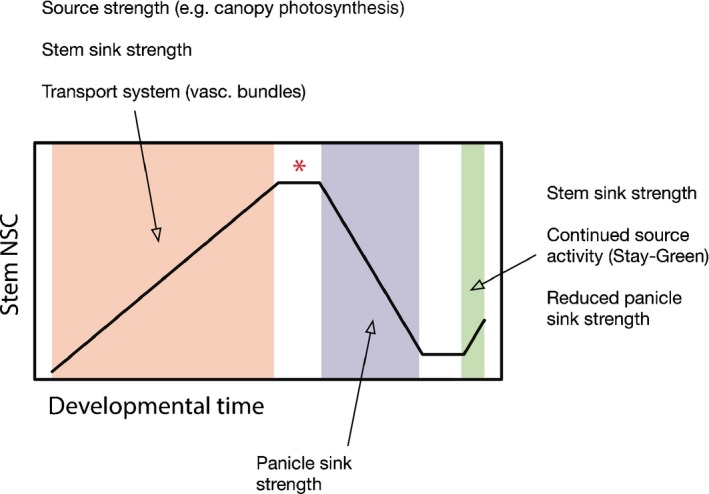
Model of rice stem nonstructural carbohydrates (NSC) throughout development. Overview of net stem NSC dynamics and forces that theoretically influence these levels. Orange, period of net NSC accumulation; purple, period of net loss; green, end‐of‐season reaccumulation of stem NSCs, influenced by G, E and G × E (G, Genotype; E, Environment; G × E, Genotype by Environment interaction). Red asterisk marks heading.

The processes that constrain and enable the dynamics of rice stem NSC are complex: preheading accumulation is likely influenced by source strength of photosynthetic leaves and sink strength of stems; post‐heading remobilization is hypothesized to be panicle sink‐driven; and, finally, end‐of‐season reaccumulation may be a function of stem sink strength, continued source activity, and reduced panicle sink capacity (Fig. 6). Morphometric variation may additionally influence efficiency or activity of sink, source and transport systems (Pan *et al*., 2011). Here, we suggest that *OsHXK6*, a hexokinase, and a tandem array of monosaccharide transporters could underlie QTL found on chromosomes 1 and 11 and associated with starch‐at‐maturity and TNC‐at‐heading, respectively. Having both catalytic and sugar‐sensing roles, hexokinases have been called the ‘gateway of glucose metabolism’ (Frommer *et al*., 2003). Modification of *OsHXK6* or its expression may potentially alter carbon metabolism, resulting in observed phenotypic differences in stem carbohydrates. So far we do not know conclusively whether source activity of flag leaf photosynthesis, sink strength of stems or variation in transport efficiency from leaf to stem represents the limiting factor in pre‐heading stem NSC accumulation in rice; the primary constraint may differ across genotypes and environments. Changes to the functionality of the four tandemly arrayed MSTs on chromosome 11 could plausibly affect transportation of hexoses and pentoses (basic units of carbon metabolism) and lead to alteration of stem NSC accumulation efficiency.

In the present study, we also point to ISA2 (*OsIsa2*) as a potential candidate underlying a sucrose‐at‐maturity QTL. Isoamylases are a type of debranching enzyme that function in plants to mediate proper amylopectin formation, but their specific roles are species‐ and organ‐dependent. The classic response of isoamylase disruption is the accumulation of soluble phytoglycogen in lieu of insoluble amylopectin, best studied in the *sugary1* maize mutants (Lin *et al*., 2013). However, the extent of this phenotype is highly dependent upon allelic variation. In rice, there are two isoamylase protein oligomers: an ISA1 homo‐oligomer shown to be unstable at high temperature (40°C) and an ISA1‐ISA2 hetero‐oligomer that is stable at high temperature (Utsumi & Nakamura, 2006). The relative abundance and functions of the oligomers differ by tissue location. While both homo‐ and hetero‐oligomers are present, just the ISA1 homo‐oligomer is essential for amylopectin production in the rice endosperm (Utsumi *et al*., 2011). By contrast, only the hetero‐oligomer is present and required in the rice leaf. It has been hypothesized that a fundamental divergence in these organs’ roles as storage locations underlie their contrasting isoamylase requirements: the leaf has a diurnal starch cycling pattern throughout the entirety of a plant's life while the endosperm represents one‐time storage to provide energy for germination (Utsumi *et al*., 2011). ISA1 expression is many‐fold higher than ISA2 in the endosperm, while ISA2 expression is greater than ISA1 in the leaf (Ohdan *et al*., 2005). Whether rice stem starch is more akin to assimilatory starch of the leaf or reserve starch of the endosperm remains to be determined. If the QTL on chromosome 5 continues to fine map to *OsIsa2*, it would be of interest to address the following points: determine the relationship between stem sucrose‐at‐maturity levels and stem starch formation; analyze which oligomers are present in the rice stem and which are essential for normal starch formation; investigate *OsIsa1 and OsIsa2* transcriptional patterns in the rice stem throughout the growth period; characterize the relative abundance and distribution of glucans (amylose, amylopectin, phytoglycogen) in lines that carry contrasting variants of the QTL.

One curious observation from our results is that increased stem NSC, regardless of the specific trait (starch, sucrose, TNC) or developmental point (heading, maturity) was consistently associated with the minor allelic state, that is, the rare allele in *tropical japonica* (Fig. S12) that was found more frequently in other subpopulations (*indica*,* aus* and *aromatic*) (Tables S15, S21). For the chromosome 5 QTL associated with sucrose‐at‐maturity that was discovered in US‐TRJ, only six accessions carried the minor allele, out of which five (cv Carolina Gold, Blue Rose, Blue Rose Supreme, Honduras and Lady Wright Selection) were directly selected from the earliest introductions into the United States before 1930 and have unknown pedigrees. They were subsequently used as parents in some of the earliest crosses in the history of US rice breeding (Figs S15, S16) (Lu *et al*., 2005), but this particular allele on chromosome 5 was lost over time, such that it was not transmitted into the gene pool of modern rice varieties. In regions along the Gulf Coast in the US, rice is often ratooned to produce a second crop, increasing annual crop yield per unit land area with minimal additional input (e.g. fertilizer, labor). Ability to yield a good ratoon crop has been associated with nonstructural carbohydrate reserve levels remaining in the stubble after main crop harvest; these NSCs may provide energy for new tiller growth (Vergara *et al*., 1988). Despite obvious economic benefits that can be gained from producing a second crop, breeding programs only evaluate ratooning ability in late generations when breeding lines are nearly fixed genetically due to the additional time and labor costs associated with early generation screening. In light of this, it may be of interest to evaluate the effect of the chromosome 5 QTL, currently absent in modern varieties, on ratooning ability as a potential for early generation marker for ratooning.

In addition to identifying genomic regions associated with stem NSC traits, this study provides an analytical pipeline for the rapid prediction of NSC traits using NIR spectral methods (Methods S2). One constraint to large‐scale genetic studies on stem NSC has been the labor associated with traditional wet chemistry assays that have been used to determine carbohydrate constituents in biomass samples. Although NIR methods have been utilized to predict water‐soluble carbohydrates in wheat (Wang *et al*., 2011) and bioenergy‐relevant carbohydrates in sorghum (Brenton *et al*., 2016), model calibration and sample prediction are typically performed using proprietary software; some common industry standards are The Unscrambler (CAMO Software AS) and WinISI (FOSS). Here, rather than using commercial packages, we generated predictions on our GLOBAL‐TRJ panel using only publically available R packages and custom‐written R scripts. These resources are linked into a coherent pipeline as Supporting Information (Methods S2). Although additional refinement will be needed before generalizing to other studies, this information can serve as an initial framework for the greater plant community to utilize in studying diverse biomass samples from other species, organs and sampling points, conditional on suitable NIR calibration sets.

Continued investigation is needed to understand the molecular mechanisms and dynamic regulatory signals governing the synthesis, storage, breakdown and remobilization of NSC within a plant and to evaluate whether QTL will be detected and useful under field conditions. The development of high‐throughput phenotyping techniques, rapid advancements in computing, and decreasing cost of genotyping will provide hitherto unexplored opportunities to learn about the relationship between genetic variation and stem carbohydrate plasticity, and we may one day be able to harness the potential of stem NSC for enhancing rice yield stability in the face of changing and highly variable climatic conditions.

## Author contributions

D.R.W., E.J.W. and S.R.M. designed research; D.R.W. and R.H. collected data; D.R.W., E.J.W. and S.R.M. analyzed and interpreted data; and D.R.W. and S.R.M. wrote the manuscript.

## Supporting information

Please note: Wiley Blackwell are not responsible for the content or functionality of any Supporting Information supplied by the authors. Any queries (other than missing material) should be directed to the *New Phytologist* Central Office.

**Fig. S1** Stem samples used for near‐infrared spectroscopy model calibration.
**Fig. S2** Scree plot of PCA on US‐TRJ panel using 38 618 SNPs.
**Fig. S3** LD of US‐TRJ vs GLOBAL‐TRJ.
**Fig. S4** Quantile–quantile plots from stem NSC GWAS on US‐JAPONICA and US‐TRJ panels.
**Fig. S5** Local LD decay around msSNPs of GLOBAL‐TRJ QTL.
**Fig. S6** Transcriptional information of putative candidate genes underlying stem NSC QTL identified from GWAS.
**Fig. S7** Stem NSC distributions across market classes in US rice.
**Fig. S8** Investigation into the effect of days to heading on stem NSC GWAS results.
**Fig. S9** Local LD heatmaps of significant sub‐QTL regions at the chromosome 1 of GLOBAL‐TRJ.
**Fig. S10** GWAS results for NSC traits in GLOBAL‐TRJ.
**Fig. S11** LD plot of chromosome 11 of a region associated with starch‐at‐heading.
**Fig. S12** Phenotypic distributions grouped by genotype class for associated traits of two significant peaks found from both US‐TRJ and GLOBAL‐TRJ GWAS.
**Fig. S13 **Phenotypic distributions of genotypes harboring different combinations of alleles of the msSNPs from chromosomes 1 and 11 QTL.
**Fig. S14** Sequence polymorphisms cataloged in LOC_Os05g32710, isoamylase.
**Fig. S15** Distribution of chromosome 5 msSNP alleles across US rice.
**Fig. S16** Haplotype analysis of chromosome five QTL associated with sucrose‐at‐maturity in US‐TRJ.

**Table S1** Germplasm information
**Table S2** NSC phenotypes of US‐TRJ panel
**Table S3** NSC phenotypes of GLOBAL‐TRJ panel
**Table S4** *A priori* candidate genes
**Table S5** GWAS QTL from US‐TRJ panel
**Table S6** Heritability of NIR predicted NSC traits
**Table S7** GWAS results from GLOBAL‐TRJ panel
**Table S8** SNPs and indels in *OsHXK6*
**Table S9** Gene models underlying chromosome 1 QTL (Fig. 3d)
**Table S10** Gene models underlying chromosome 11 QTL (Fig. 4d)
**Table S11** SNPs and indels in LOC_Os11g41830
**Table S12** SNPs and indels in LOC_Os11g41840
**Table S13** SNPs and indels in LOC_Os11g41850
**Table S14** SNPs and indels in LOC_Os11g41870
**Table S15** Allele distribution of msSNPs from chromosome 1 and 11 QTLs across diverse *Oryza sativa* accessions
**Table S16** IciMapping results on Curinga CSSL panel
**Table S17** Graphical genotype of Curinga, IRGC105491, and the backcross progeny between CURUF25 and Curinga
**Table S18** SNPs and indels in *OsIsa2*
**Table S19** US‐TRJ GWAS results: −log_10_(*P*‐value)
**Table S20** GLOBAL‐TRJ GWAS results: −log_10_(*P‐*value). Note that markers that did not pass the 0.05 minor allele frequency cut‐off have a −log_10_(*P*‐value) of 0
**Table S21** Allele counts of SNP S5_19425787 across diverse *Oryza sativa* subpopulation control samples

**Methods S1** CSSL IciMapping input file with genotype and phenotype information.

**Methods S2** Zip file that contains full description of NIR prediction analysis, spectral data file, and relevant code.
