# Supplementary material for: The buffering capacity of stems: genetic architecture of nonstructural carbohydrates in cultivated Asian rice, Oryza sativa
Source: New Phytol. 2017 May 30;215(2):658–71. doi: 10.1111/nph.14614 (PMC5488208; doi:10.1111/nph.14614)
Supplement: Supplementary file 1 — Fig. S1 Stem samples used for near‐infrared spectroscopy model calibration. Fig. S2 Scree plot of PCA on US‐TRJ panel using 38 618 SNPs. Fig. S3 LD of US‐TRJ vs GLOBAL‐TRJ. Fig. S4 Quantile–quantile plots from stem NSC GWAS on US‐JAPONICA and US‐TRJ panels. Fig. S5 Local LD decay around msSNPs of GLOBAL‐TRJ QTL. Fig. S6 Transcriptional information of putative candidate genes underlying stem NSC QTL identified from GWAS. Fig. S7 Stem NSC distributions across market classes in US rice. Fig. S8 Investigation into the effect of days to heading on stem NSC GWAS results. Fig. S9 Local LD heatmaps of significant sub‐QTL regions at the chromosome 1 of GLOBAL‐TRJ. Fig. S10 GWAS results for NSC traits in GLOBAL‐TRJ. Fig. S11 LD plot of chromosome 11 of a region associated with starch‐at‐heading. Fig. S12 Phenotypic distributions grouped by genotype class for associated traits of two significant peaks found from both US‐TRJ and GLOBAL‐TRJ GWAS. Fig. S13 Phenotypic distributions of genotypes harboring different combinations of alleles of the msSNPs from chromosomes 1 and 11 QTL. Fig. S14 Sequence polymorphisms cataloged in LOC_Os05g32710, isoamylase. Fig. S15 Distribution of chromosome 5 msSNP alleles across US rice. Fig. S16 Haplotype analysis of chromosome five QTL associated with sucrose‐at‐maturity in US‐TRJ. [file NPH-215-658-s001.pdf]

New Phytologist **Supporting Information**

Article title: The buffering capacity of stems: genetic architecture of stem carbohydrates in cultivated Asian rice, *Oryza sativa* L.

Authors: Diane R. Wang, Rongkui Han, Edward J. Wolfrum, Susan R. McCouch
Article acceptance date: 28 March 2017

The following Supporting Information is available for this article:

Fig. S1. Stem samples used for near-infrared spectroscopy model calibration.

Fig. S2. Scree plot of PCA on US-TRJ panel using 38,618 SNPs.

Fig. S3. LD of US-TRJ versus GLOBAL-TRJ.

Fig. S4. Quantile-quantile plots from stem NSC GWAS on US-JAPONICA and US-TRJ panels.

Fig. S5. Local LD decay around msSNPs of GLOBAL-TRJ QTL.

Fig. S6. Transcriptional information of putative candidate genes underlying stem NSC QTL identified from GWAS.

Fig. S7. Stem NSC distributions across market classes in U.S. rice.

Fig. S8. Investigation into the effect of days to heading on stem NSC GWAS results.

Fig. S9. Local LD heatmaps of significant sub-QTL regions at the chromosome 1 of GLOBAL-TRJ.

Fig. S10. GWAS results for NSC traits in GLOBAL-TRJ.

Fig. S11. LD plot of chromosome 11 of a region associated with starch- at-heading.

Fig. S12. Phenotypic distributions grouped by genotype class for associated traits of two significant peaks found from both US-TRJ and GLOBAL-TRJ GWAS.

Fig. S13. Phenotypic distributions of genotypes harboring different combinations of alleles of the msSNPs from chromosomes 1 and 11 QTL.

Fig. S14. Sequence polymorphisms cataloged in LOC_Os05g32710, isoamylase.

Fig. S15. Distribution of chromosome 5 msSNP alleles across U.S rice.

Fig. S16. Haplotype analysis of chromosome five QTL associated with sucrose-at-maturity in US-TRJ.

Table S1. Germplasm information.

Table S2. NSC phenotypes of US-TRJ panel.

Table S3. NSC phenotypes of GLOBAL-TRJ panel.

Table S4. *A priori* candidate genes.

Table S5. GWAS QTL from US-TRJ panel.

Table S6. Heritability of NIR predicted NSC traits.

Table S7. GWAS results from GLOBAL-TRJ panel.

Table S8. SNPs and indels in *OsHXK6*.

Table S9. Gene models underlying chromosome 1 QTL (Figure 3D).

Table S10. Gene models underlying chromosome 11 QTL (Figure 4D).

Table S11. SNPs and indels in LOC_Os11g41830.

Table S12. SNPs and indels in LOC_Os11g41840.

Table S13. SNPs and indels in LOC_Os11g41850.

Table S14. SNPs and indels in LOC_Os11g41870.

Table S15. Allele distribution of msSNPs from chromosome 1 and 11 QTLs across diverse *O. sativa* accessions.

Table S16. IciMapping results on Curinga CSSL panel.

Table S17. Graphical genotype of Curinga, IRGC105491, and the backcross progeny between CURUF25 and Curinga.

Table S18. SNPs and indels in *OsIsa2.*

Table S19. US-TRJ GWAS results: -log$_{10}$(pval).

Table S20. GLOBAL-TRJ GWAS results: -log10(pval). Note that markers that did not pass the 0.05 minor allele frequency cut-off have a -log$_{10}$(p-value) of 0.

Table S21. Allele counts of SNP S5_19425787 across diverse *Oryza sativa* subpopulation control samples.

Methods S1. CSSL IciMapping input file with genotype and phenotype information.

Methods S2. Zip file that contains full description of NIR prediction analysis, spectral data file, and relevant code.

Fig. S1 Stem samples used for near-infrared spectroscopy model calibration. A-B) Varietal group, sampling point, and storage condition are annotated for spectral projections onto PCA space and PLS model space. Storage condition "wet" refers to samples that underwent one round of drying after sampling and before grinding and subsequently stored under ambient conditions in a laboratory in the NE U.S. (i.e. humid summers) while "dry" samples underwent a second round of drying post-grinding and were stored in laboratory conditions in the arid Western U.S. All samples were scanned together on the same spectrophotometer. Major structure in PCA space of the calibration set was attributed to storage condition, however this structure was not seen when projected into the first two components of PLS space. C) Differences in storage condition was due primarily with differences in the spectrum associated with water. D) The prediction set fell between the "dry" and "wet" calibration sets in the range of 1850-2050nm associated with water and was more closely aligned with the "dry" set. E) Despite closer relationship to the "dry" set, the prediction set was better predicted using a calibration set of combined wet and dry than dry samples alone.

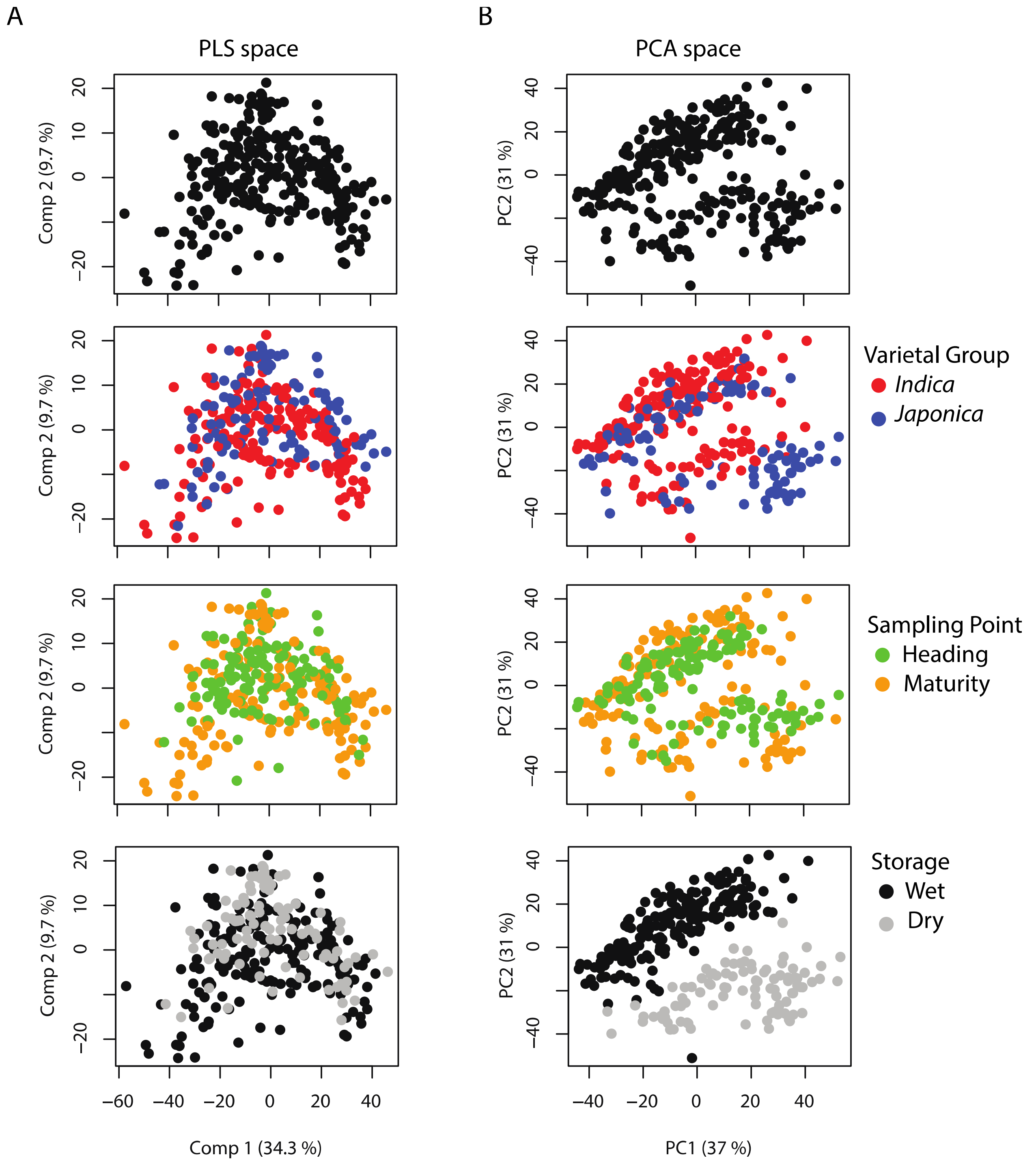
A
B
PLS space
PCA space
Comp 2 (9.7 %)
PC2 (31 %)
Varietal Group
Indica
Japonica
Sampling Point
Heading
Maturity
Storage
Wet
Dry
Comp 1 (34.3 %)
PC1 (37 %)

Fig. S1 continued.

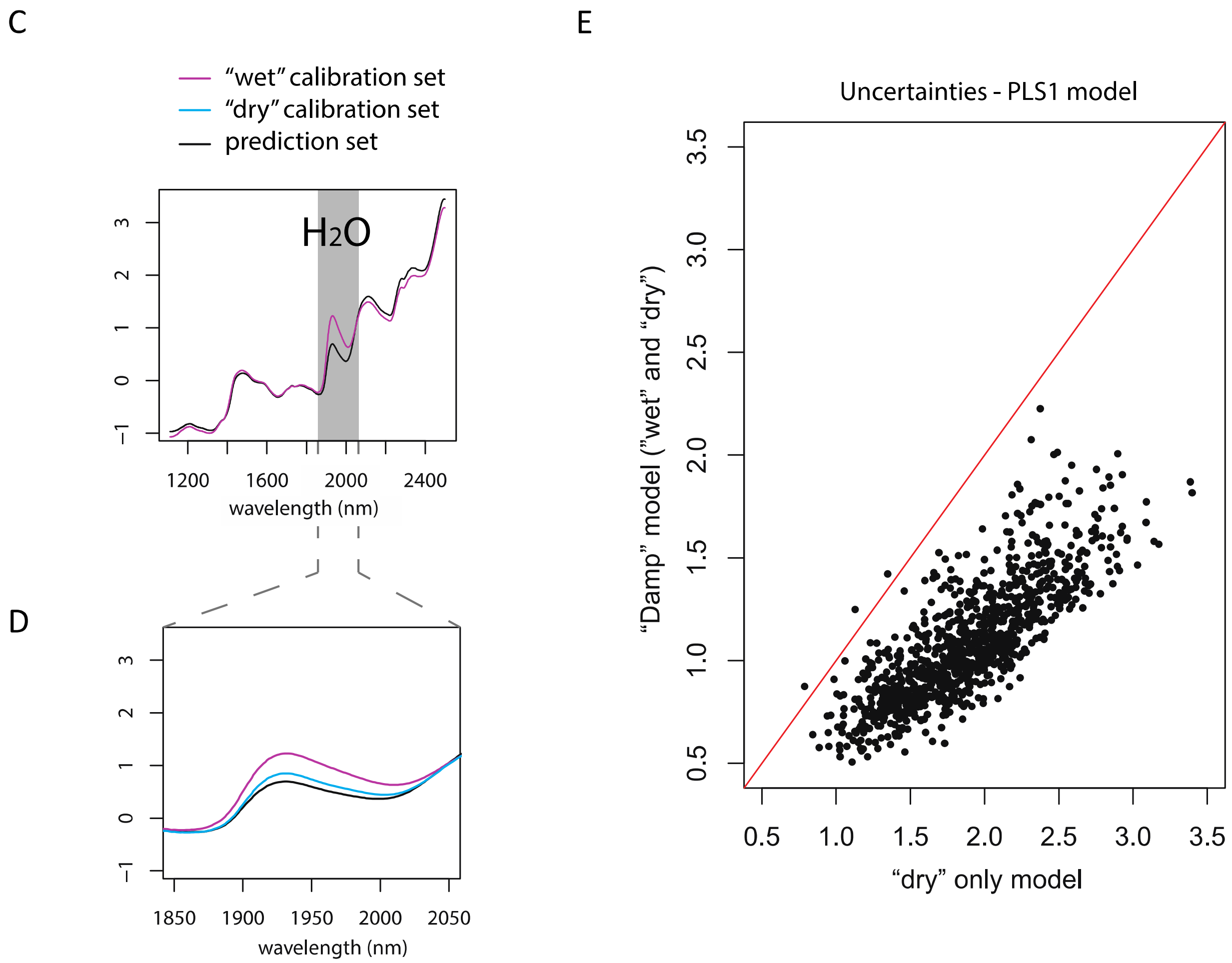

Fig. S2 Scree plot of PCA on US-TRJ panel using 38,618 SNPs.

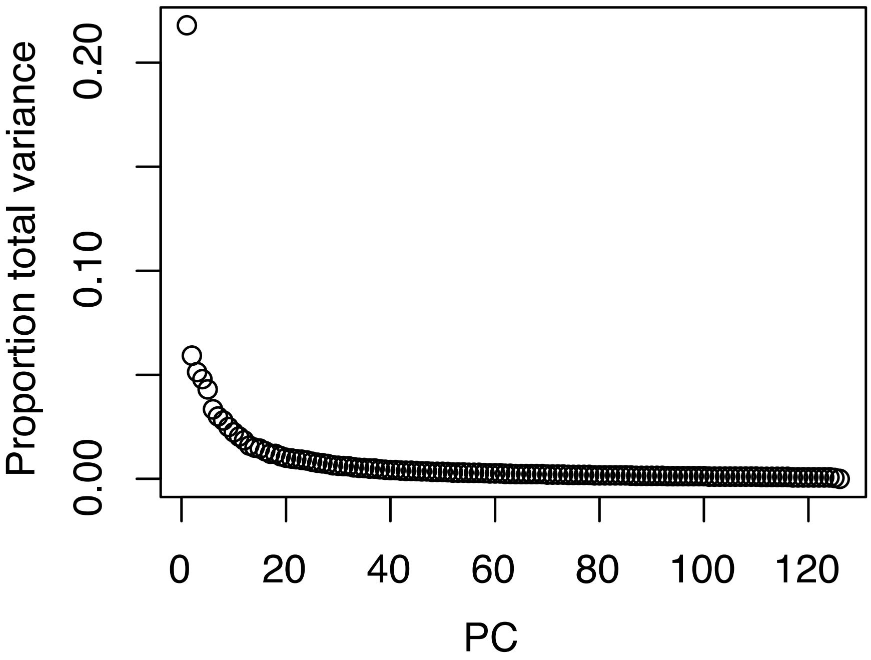

Fig. S3 LD of US-TRJ versus GLOBAL-TRJ. Pairwise LD across the rice 12 chromosomes was calculated for all SNP pairs with distances 500bp-1Mb in U.S. rice and GLOBAL-TRJ. U.S. rice shows much higher frequency of high LD SNP pairs.

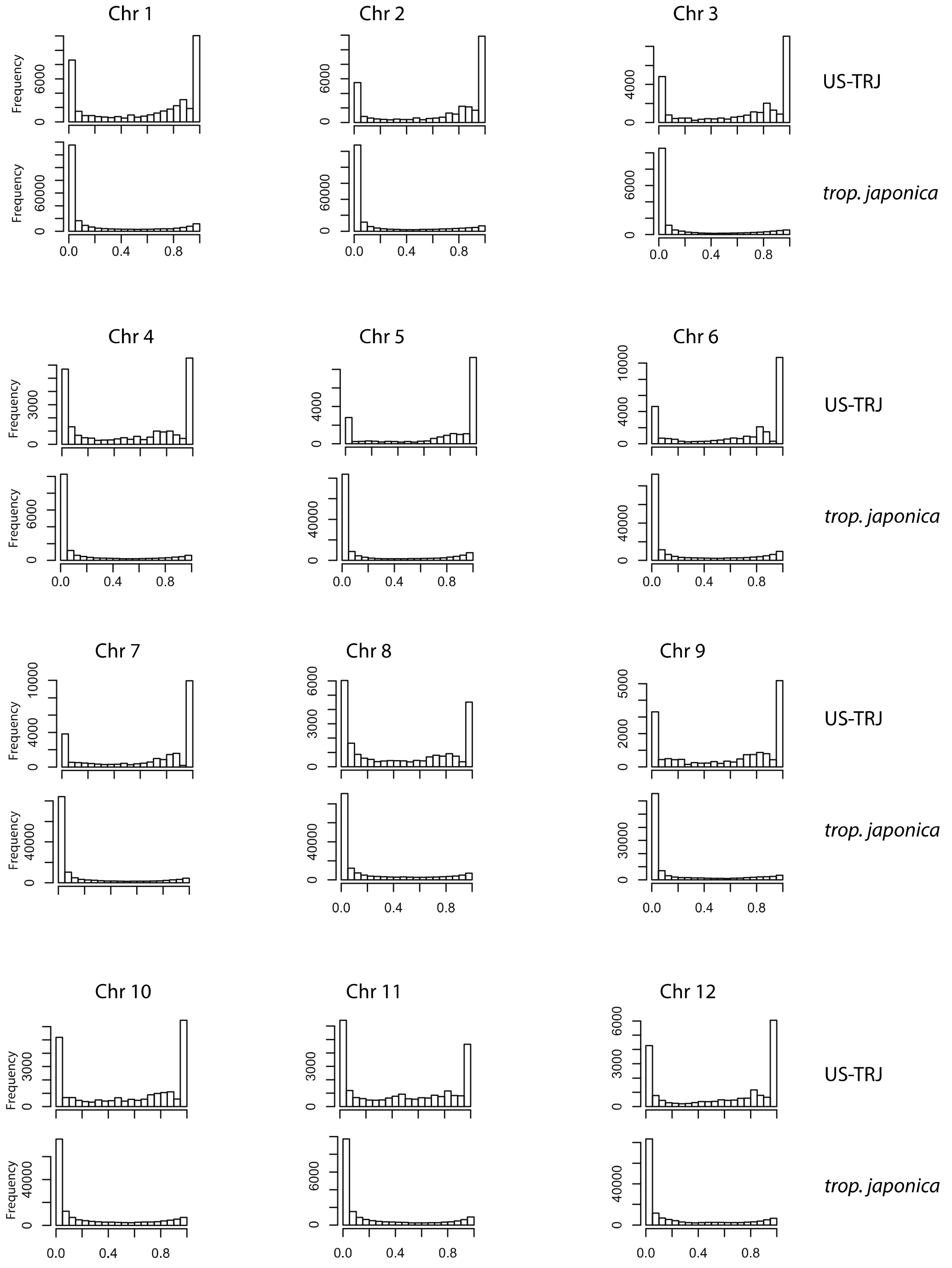

Fig. S4 Quantile-quantile plots from stem NSC GWAS on the US-TRJ panel. Red arrows indicate swaths of perfectly correlated SNPs that lead to matching p-values.

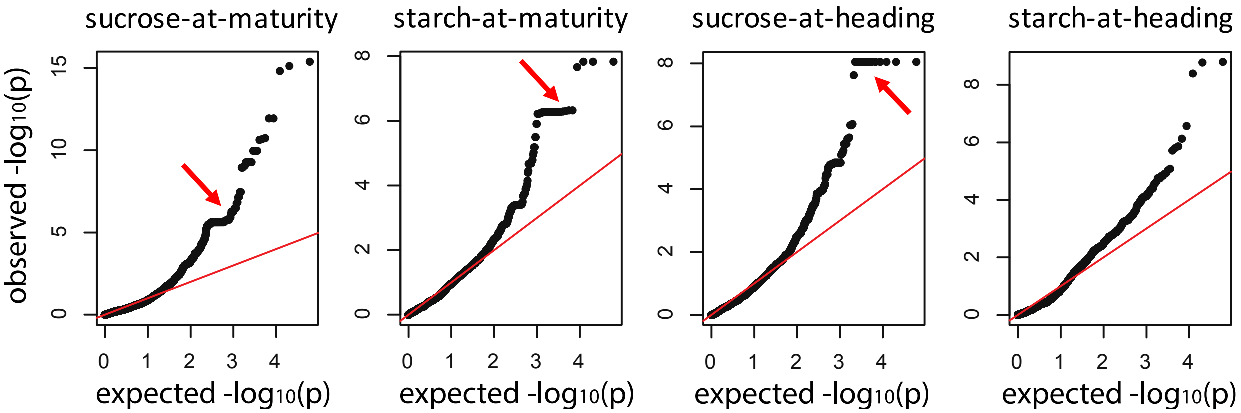

Fig. S5 Local LD decay around msSNPs of GLOBAL-TRJ QTL. SNP-specific local LD (see Materials and Methods).

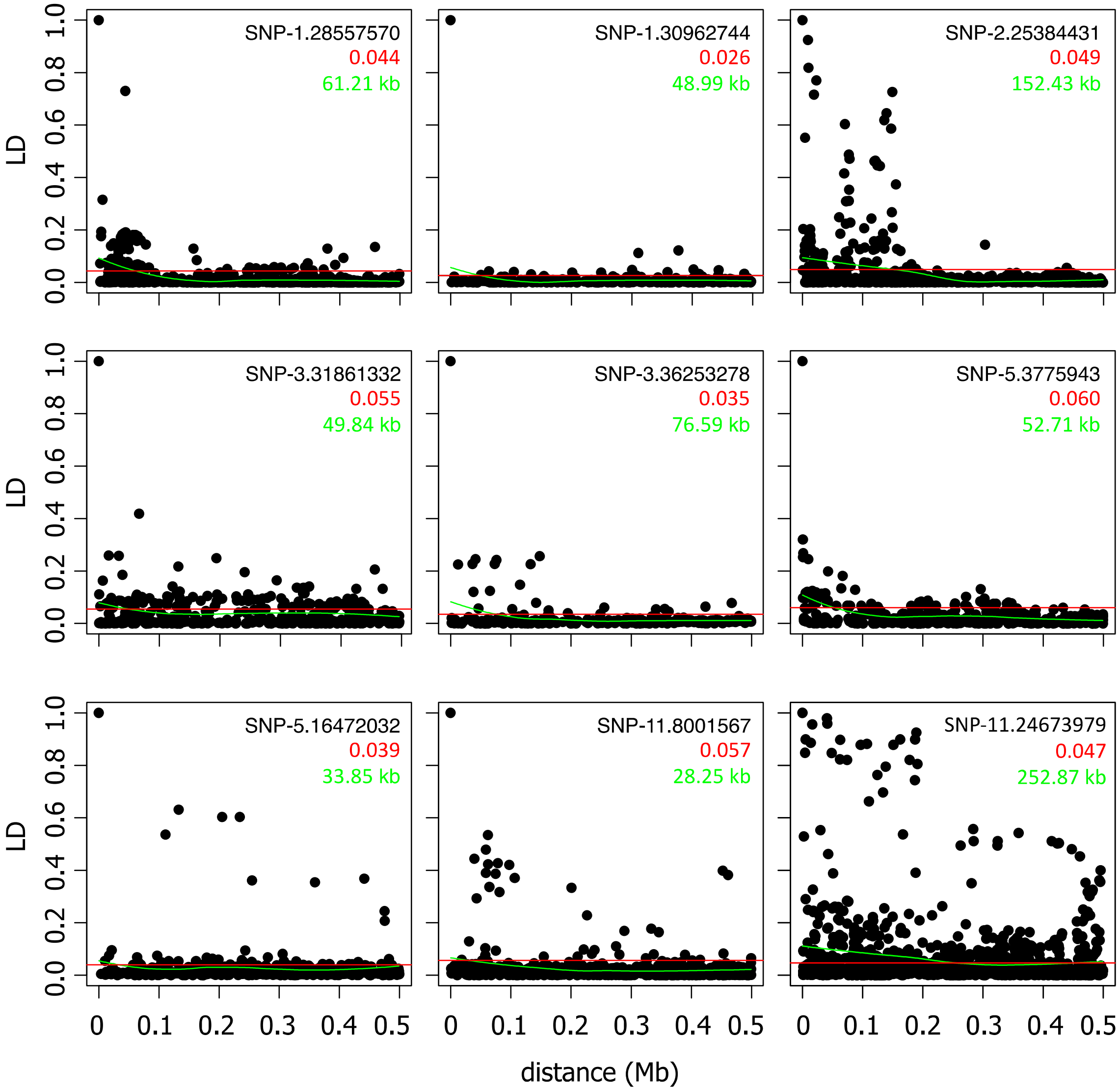

Fig. S6 Transcriptional information of putative candidate genes underlying stem NSC QTL identified from GWAS. Data were pulled from RiceXPro database (http://ricexpro.dna.affrc.go.jp/index.html) for A) LOC_Os01g53930 (*OxHXK6*), B) LOC_Os11g41830 (MST), C) LOC_Os11g41850 (MST), D) LOC_Os11g41870 (MST), and (E) LOC_Os05g32710 (*OsIsa2*). Information on LOC_Os11g41840 (MST) was unavailable.

A

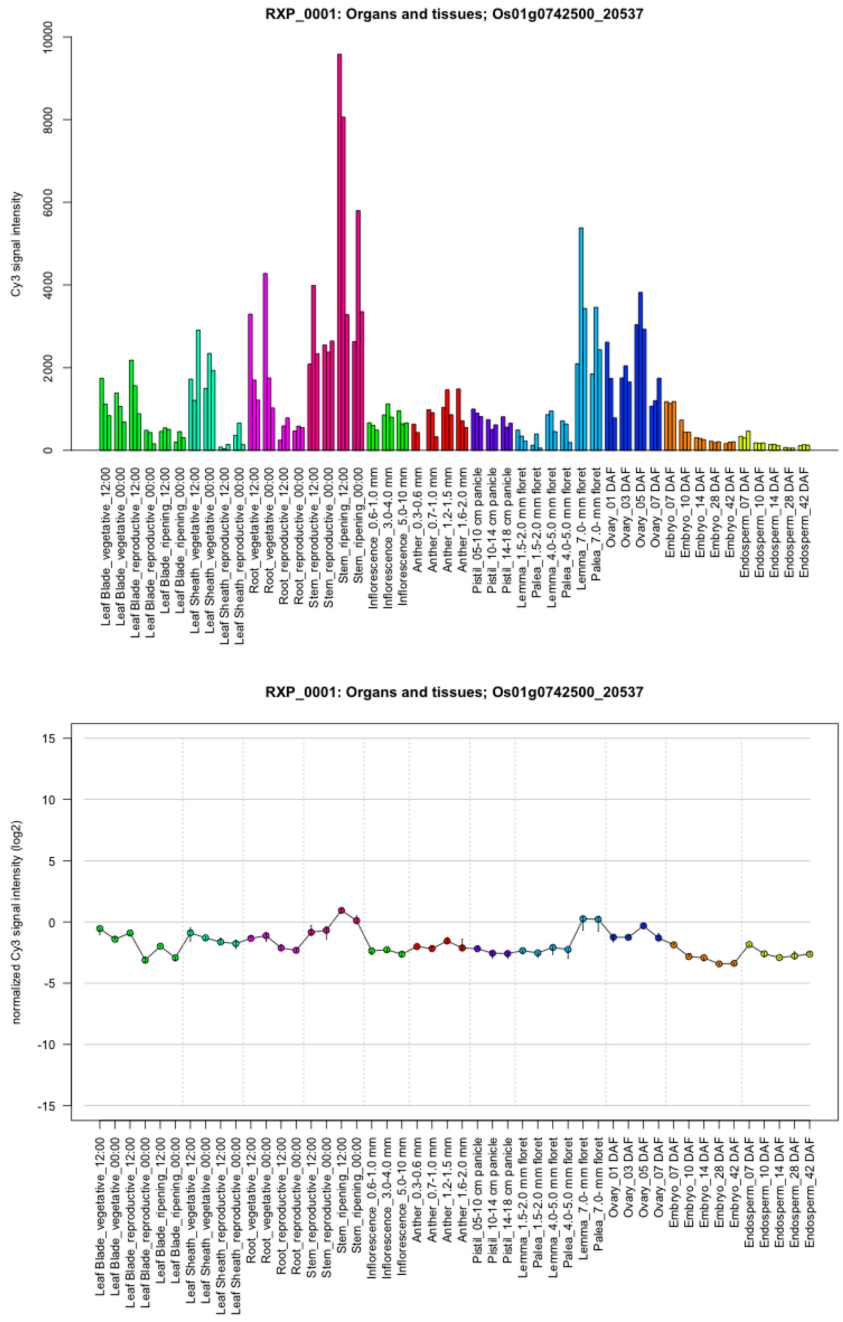

Fig. S6 continued

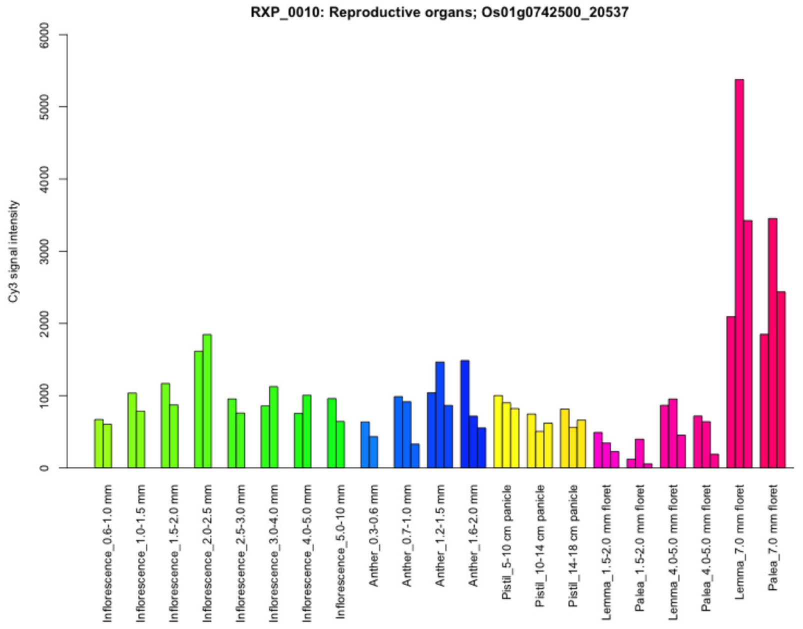

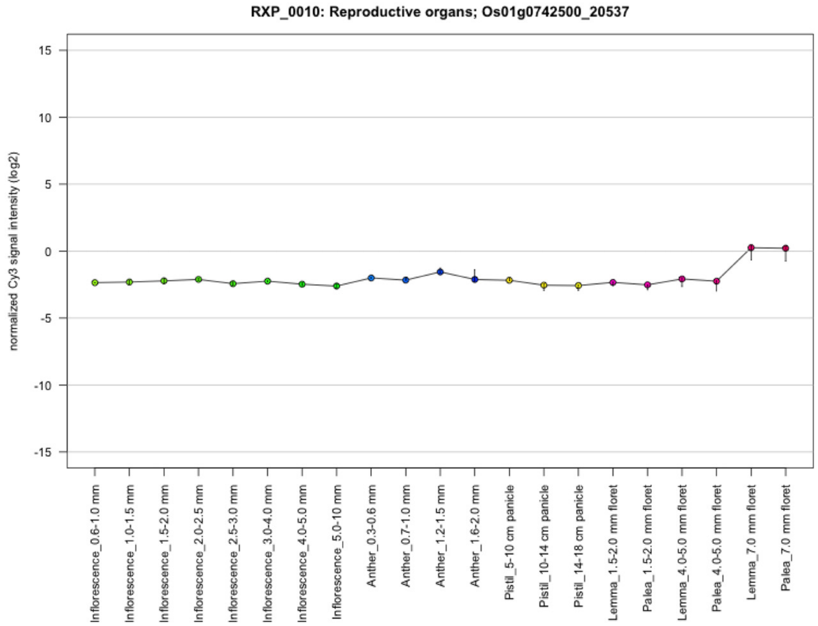

Fig. S6 continued

B

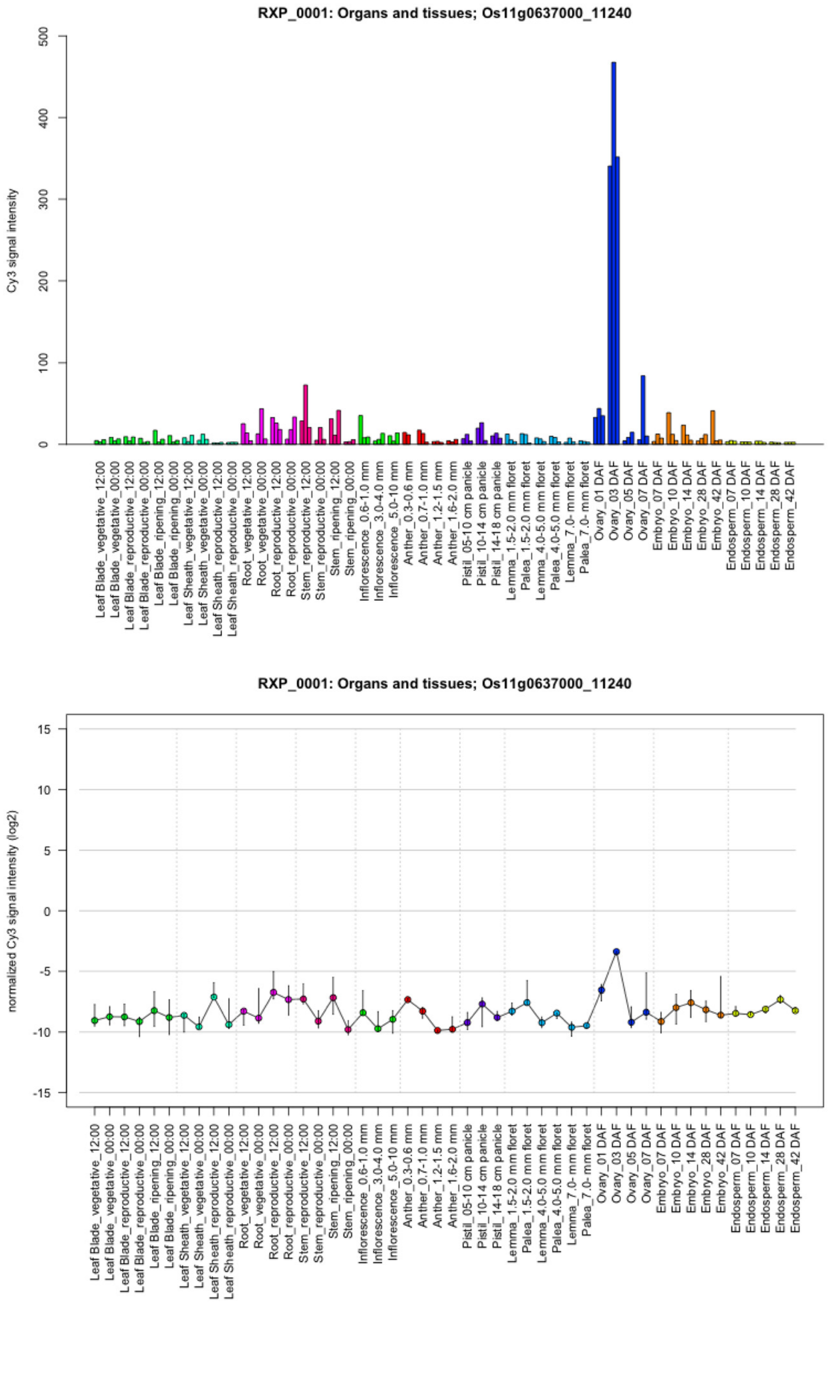

Fig. S6 continued

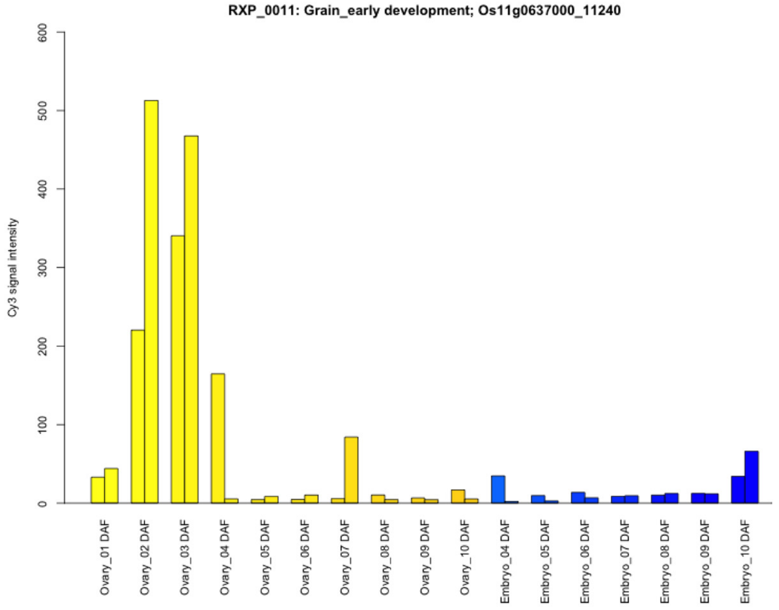

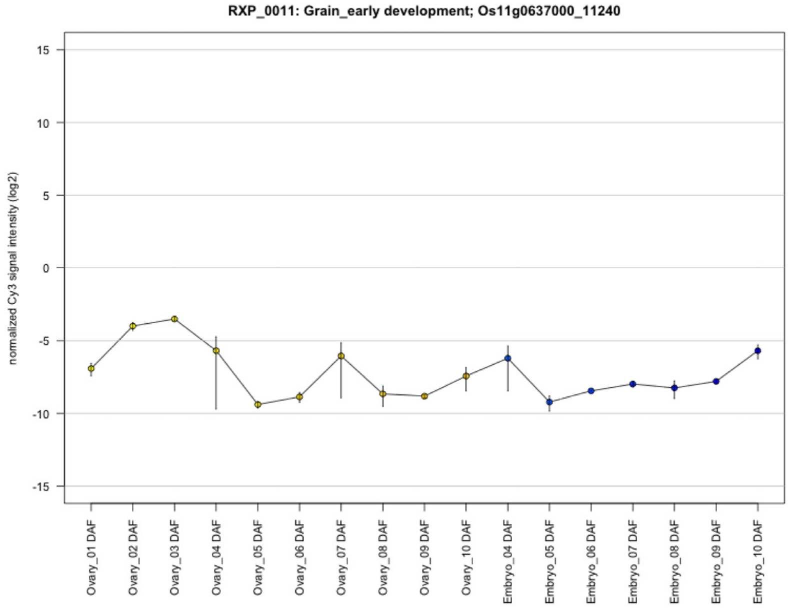

Fig. S6 continued

C

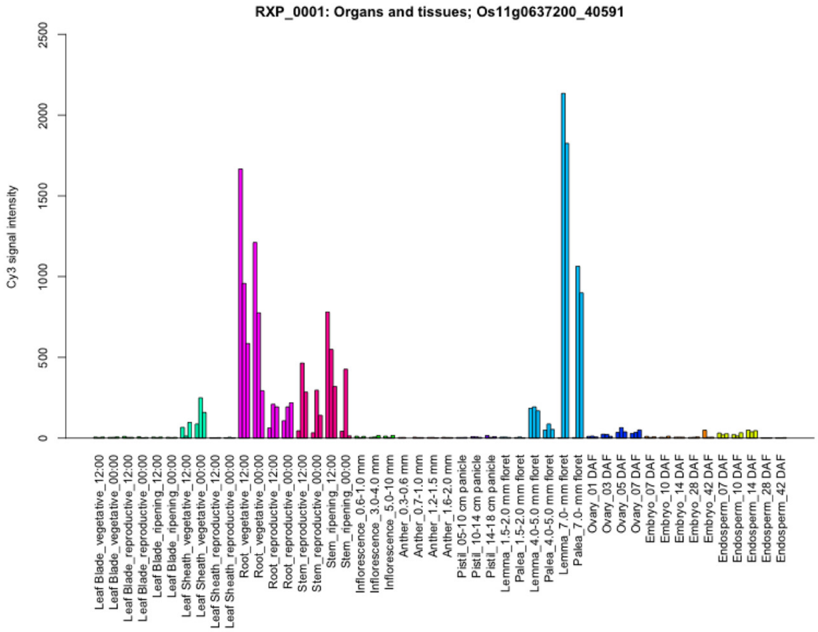

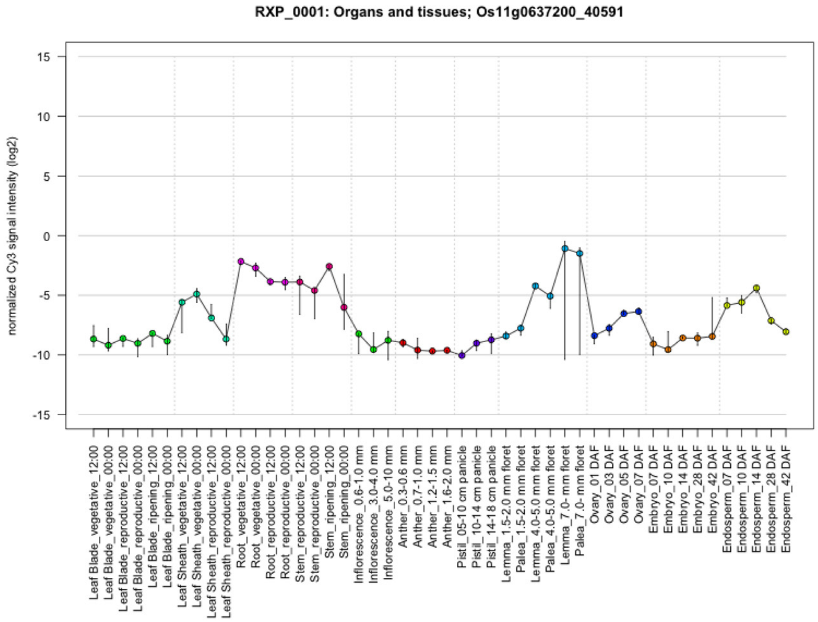

Fig. S6 continued

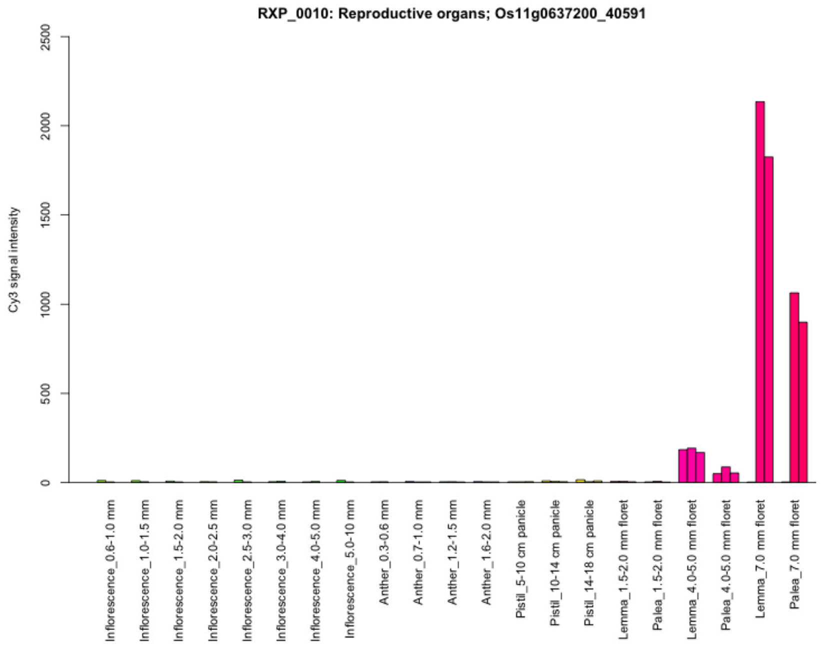

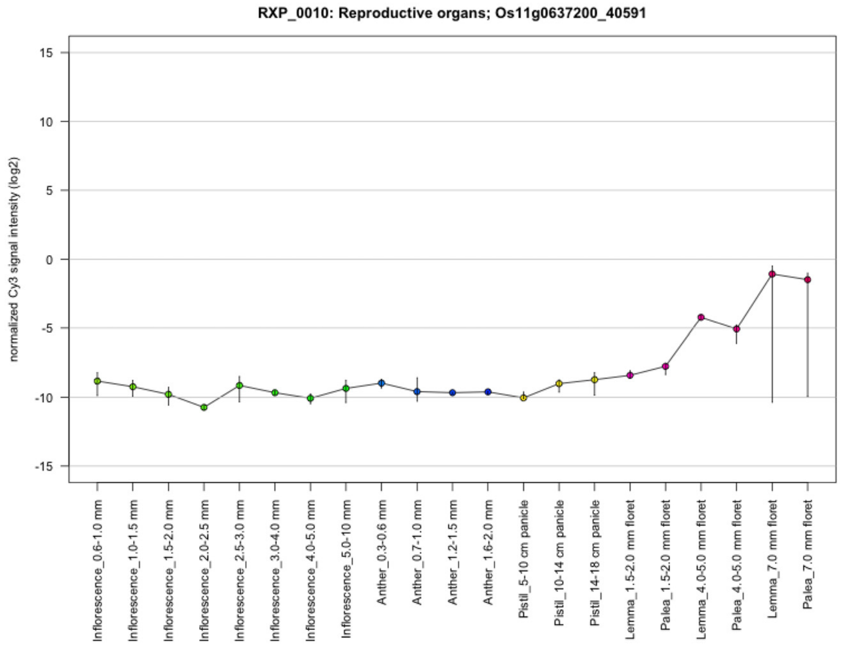

Fig. S6 continued

D

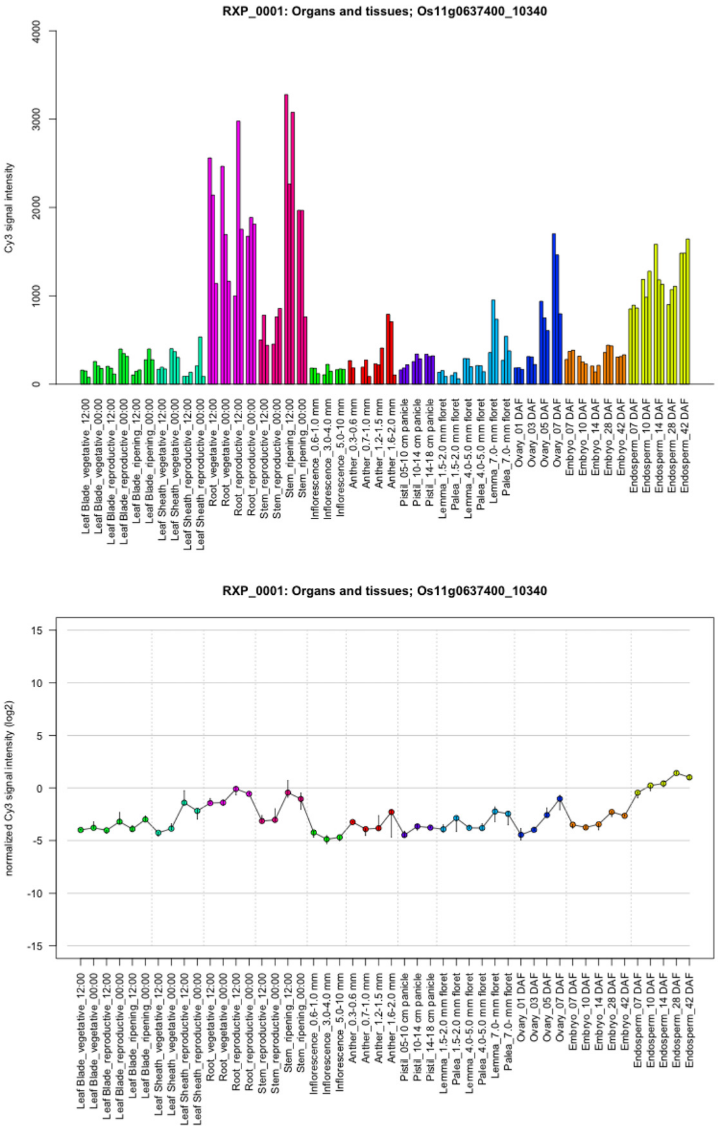

Fig. S6 continued

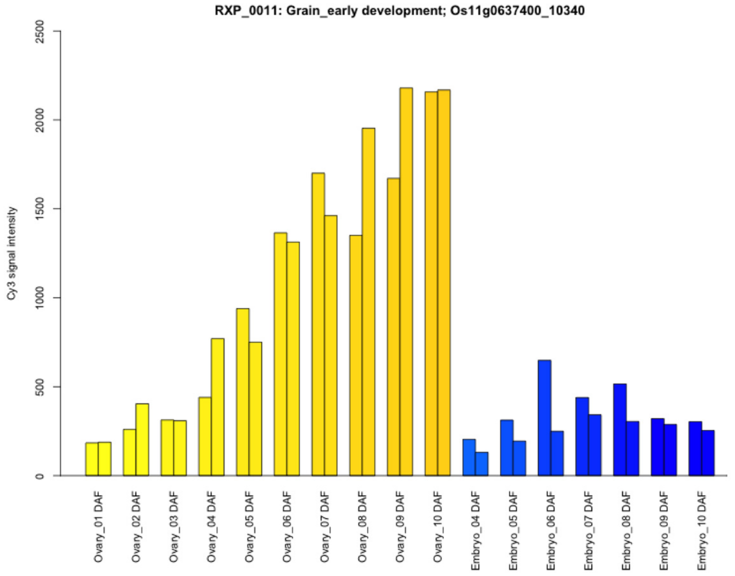

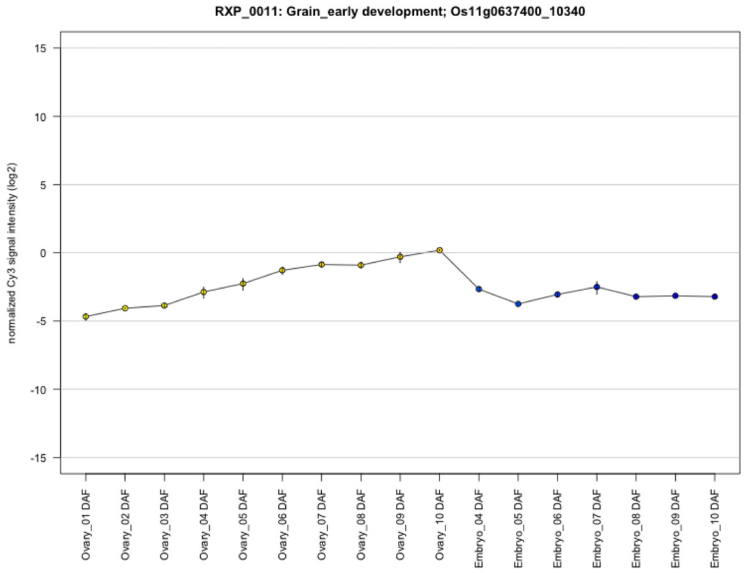

Fig. S6 continued

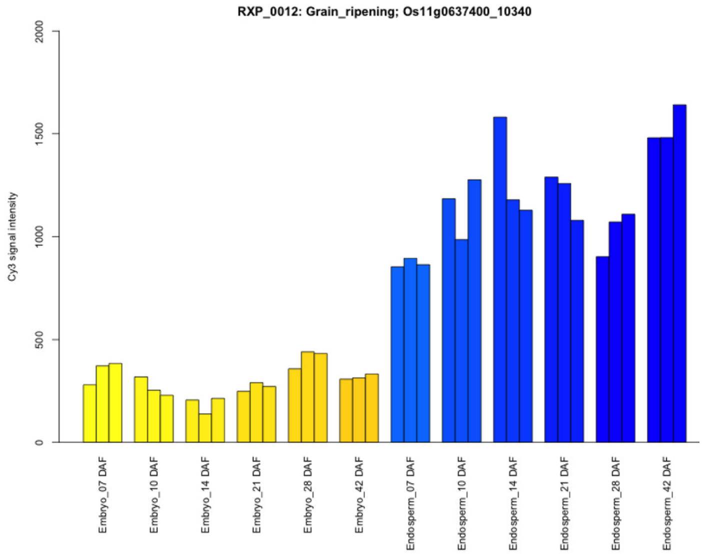

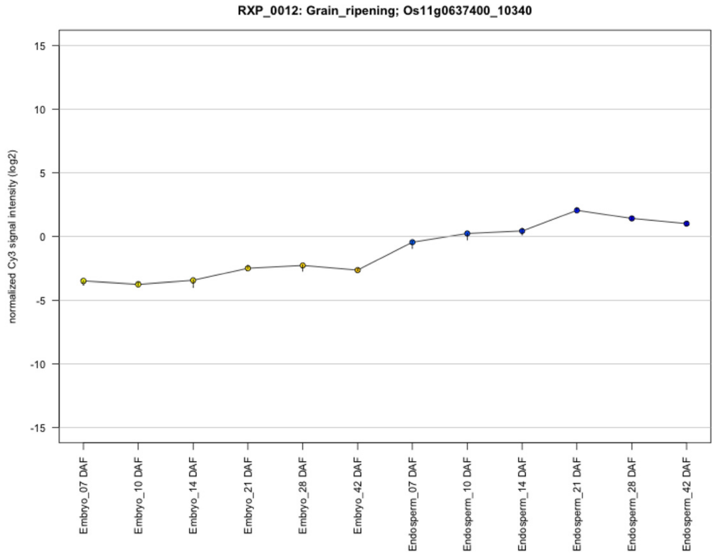

Fig. S6 continued

E

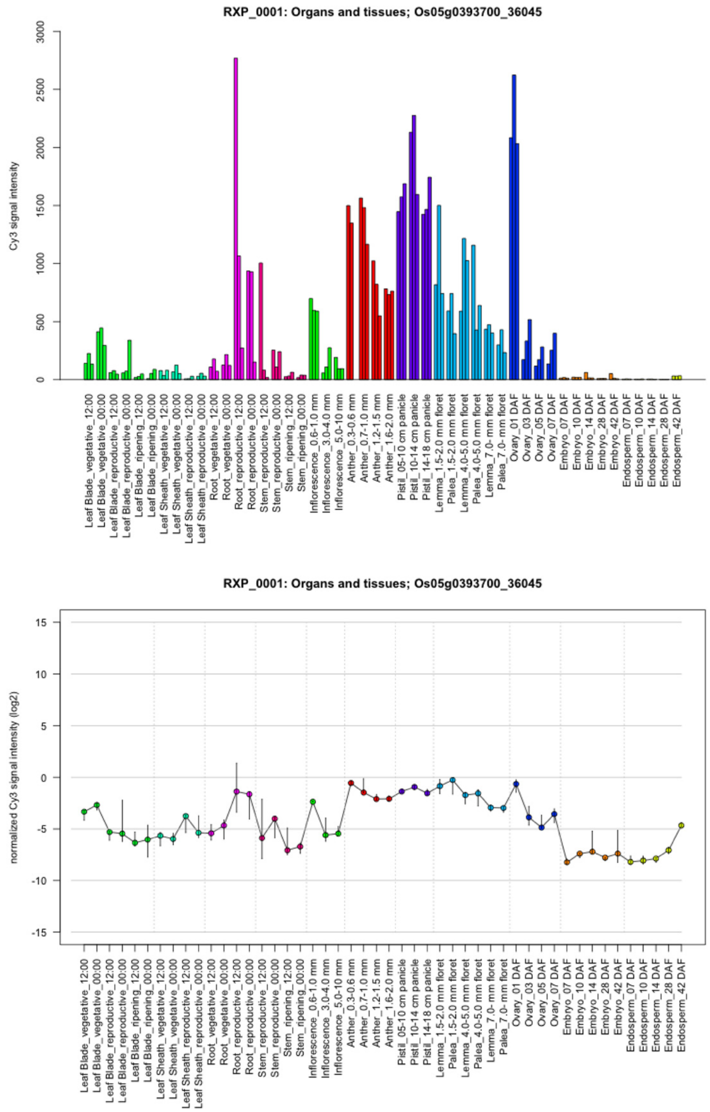

Fig. S7 Stem NSC distributions across market classes in U.S. rice. Long (n=71), medium (n=23), and short (n=2) grain length classes. ‘starch.hd.abs’ = starch-at-heading; ‘starch.mt.abs’= starch-at-maturity; ‘suc.hd.abs’= sucrose-at-heading; ‘suc.mt.abs’ = sucrose-at-maturity. Edges of boxplots are the first and third quartiles of the distributions, bars represent the highest and lowest value within the 1.5 interquartile range from the first and third quartiles, and open circles represent observations beyond those values.

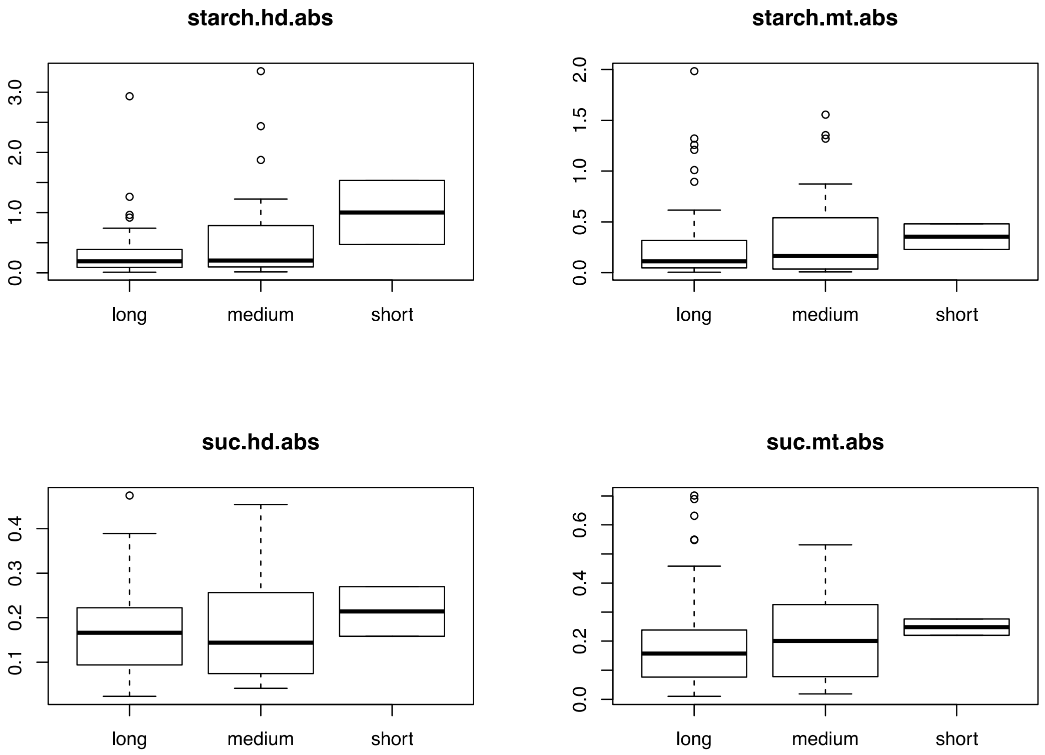

Fig. S8 Investigation into the effect of days to heading on stem NSC GWAS results. Days to heading (DTH) was incorporated as a covariate in GWAS on the TRJ-US panel for stem starch and sucrose at heading and maturity. Upper table: Correlations of resultant marker p-values from genome scans with and without DTH covariate. Lower Figure: Manhattan Plot for GWAS results of starch-at-heading with DTH covariate (0.05 FDR). Single green circle represents significant SNP.

Trait	Pearson's correlation coefficient
Starch, hd	0.77
Starch, mt	0.90
Sucrose, hd	0.93
Sucrose, mt	0.99

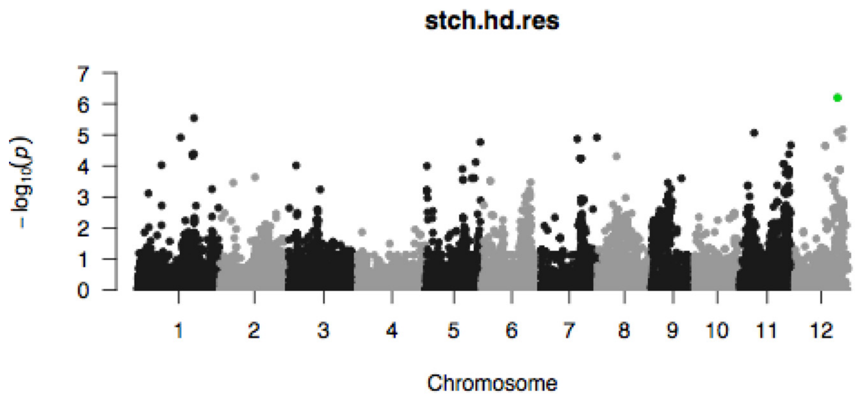

Fig. S9 Local LD heatmaps of significant sub-QTL regions at the chromosome 1 of GLOBAL-TRJ. LD (r^2) is displayed (1 = black, 0 = white, 0-1 = shades of gray). Rec circles = significant SNPs.

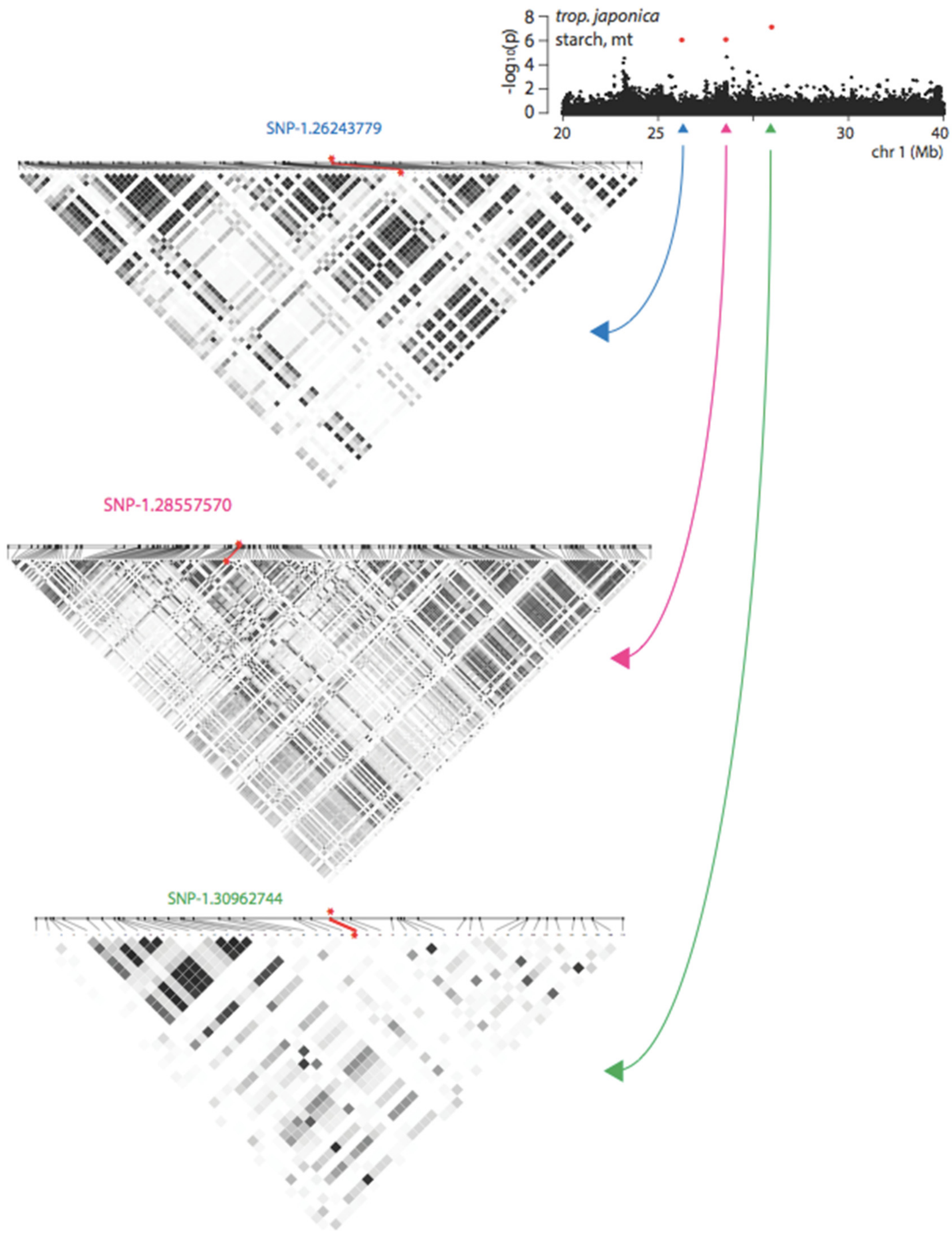

Fig. S10 GWAS results for NSC traits in GLOBAL-TRJ.

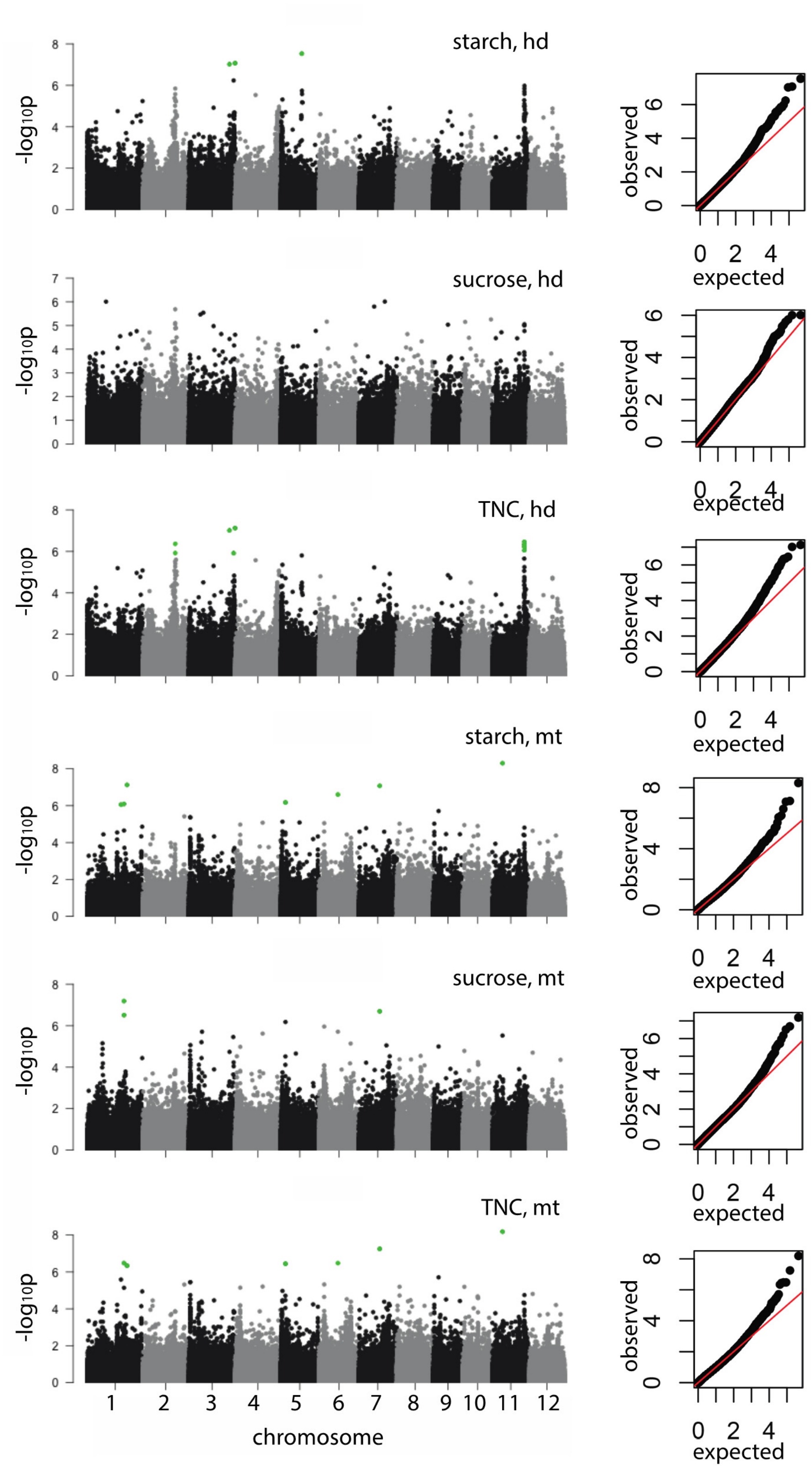

Fig. S11 LD plot of chromosome 11 of a region associated with starch- at-heading. Plot shows the region from25098867 bp to 25176821 bp. Red lines indicate positions of four significant SNPs (see Fig. 4). Top panel displays D' statistic and bottom panel displays r^2 values. Asterisk = significant SNP.

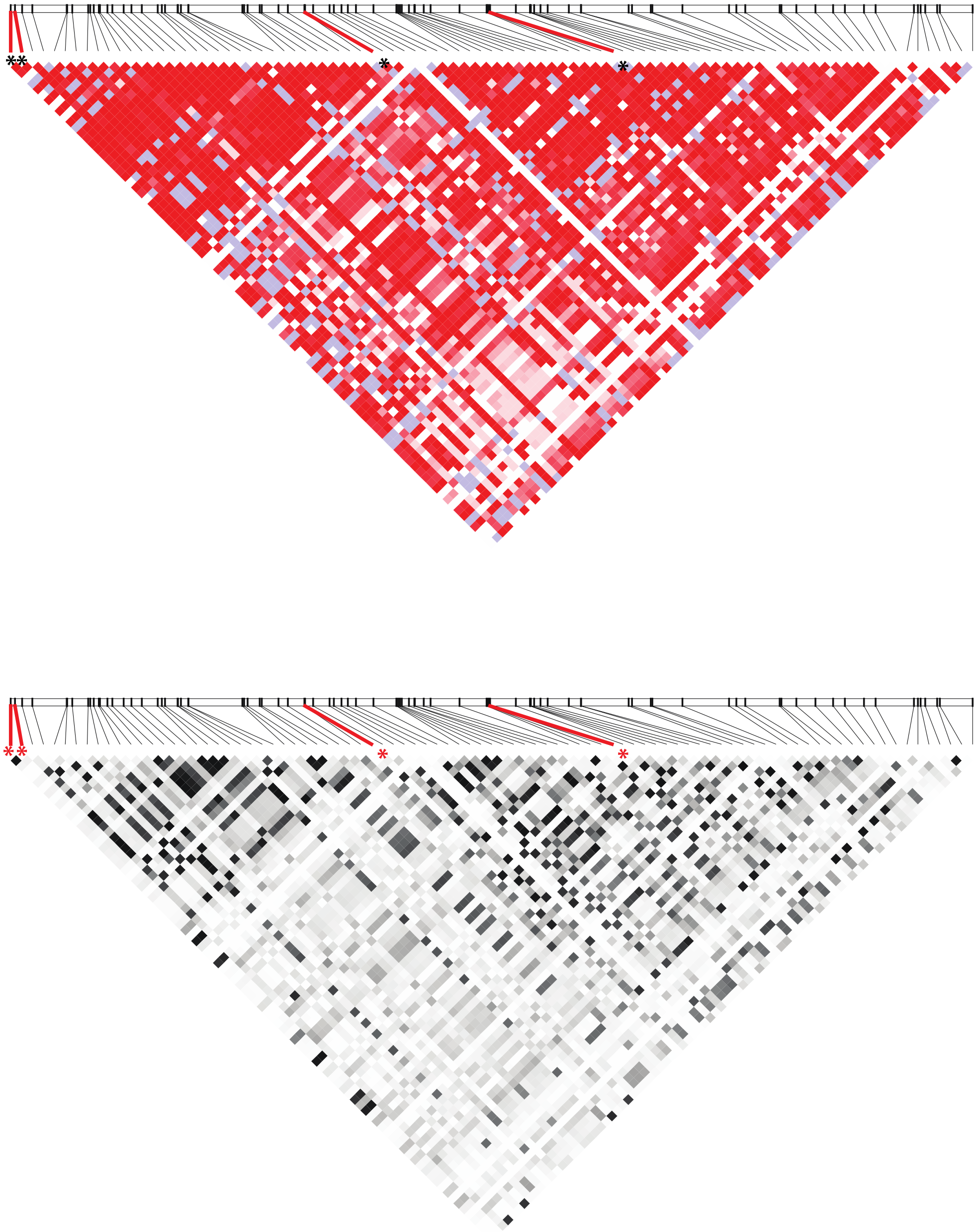

Fig. S12 Phenotypic distributions grouped by genotype class for associated traits of two significant peaks found from both US-TRJ and GLOBAL-TRJ GWAS. AA indicates the major allele and BB indicates the minor allele at each marker. Names of accession observed to carry the minor BB allele or that are heterozygous are listed below each plot in A and next to the plot in B.

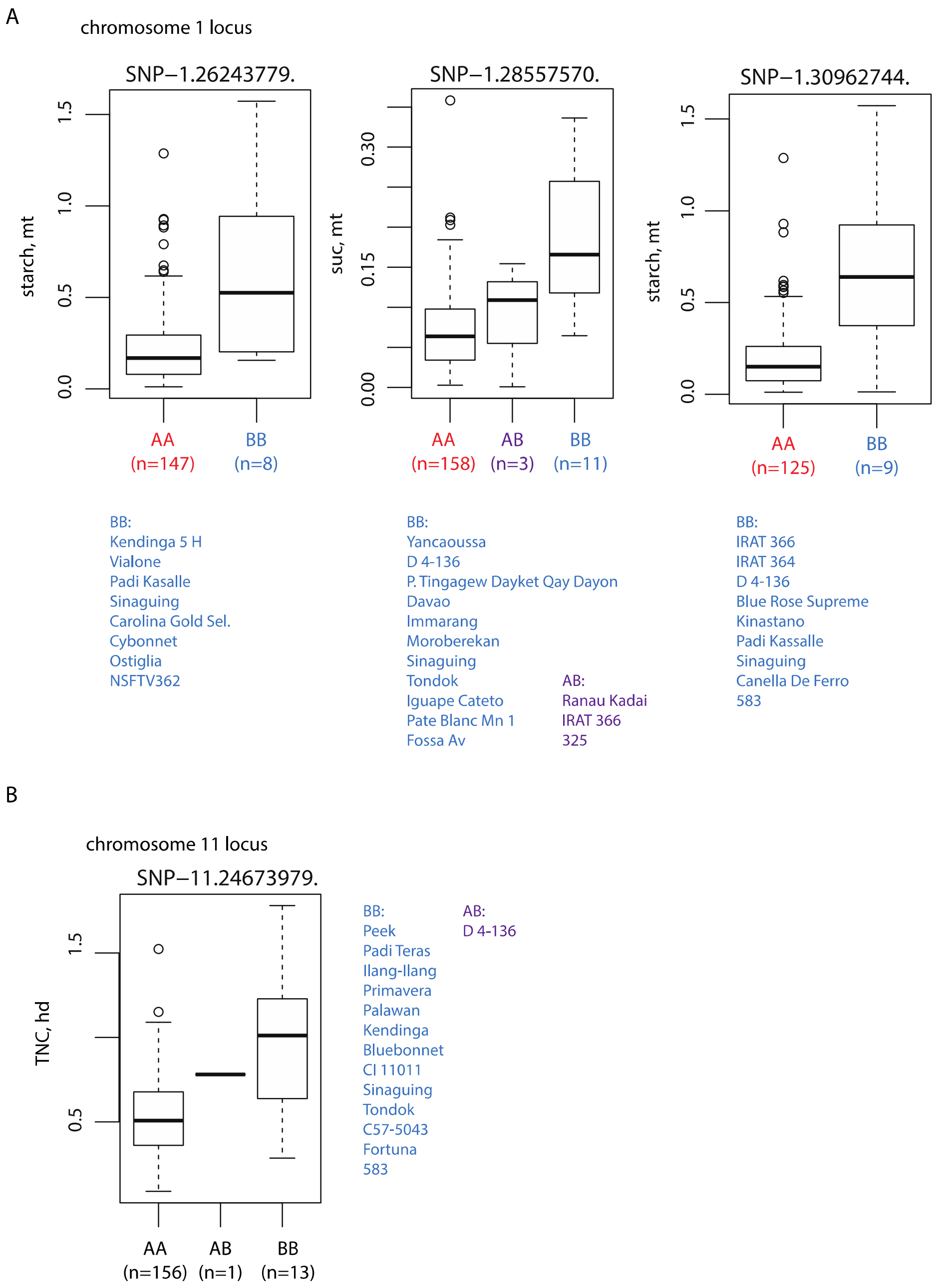

Fig. S13 Phenotypic distributions of genotypes harboring different combinations of alleles of the msSNPs from chromosomes 1 and 11 QTL. G1= chr1 major allele/chr11 major allele ; G2= chr1 major allele/chr11 minor allele; G3= chr1 minor allele/chr11 major allele; G4= chr1 minor allele/chr11 minor allele. Edges of boxplots are the first and third quartiles of the distributions, bars represent the highest and lowest value within the 1.5 interquartile range from the first and third quartiles, and open circles represent observations beyond those values.

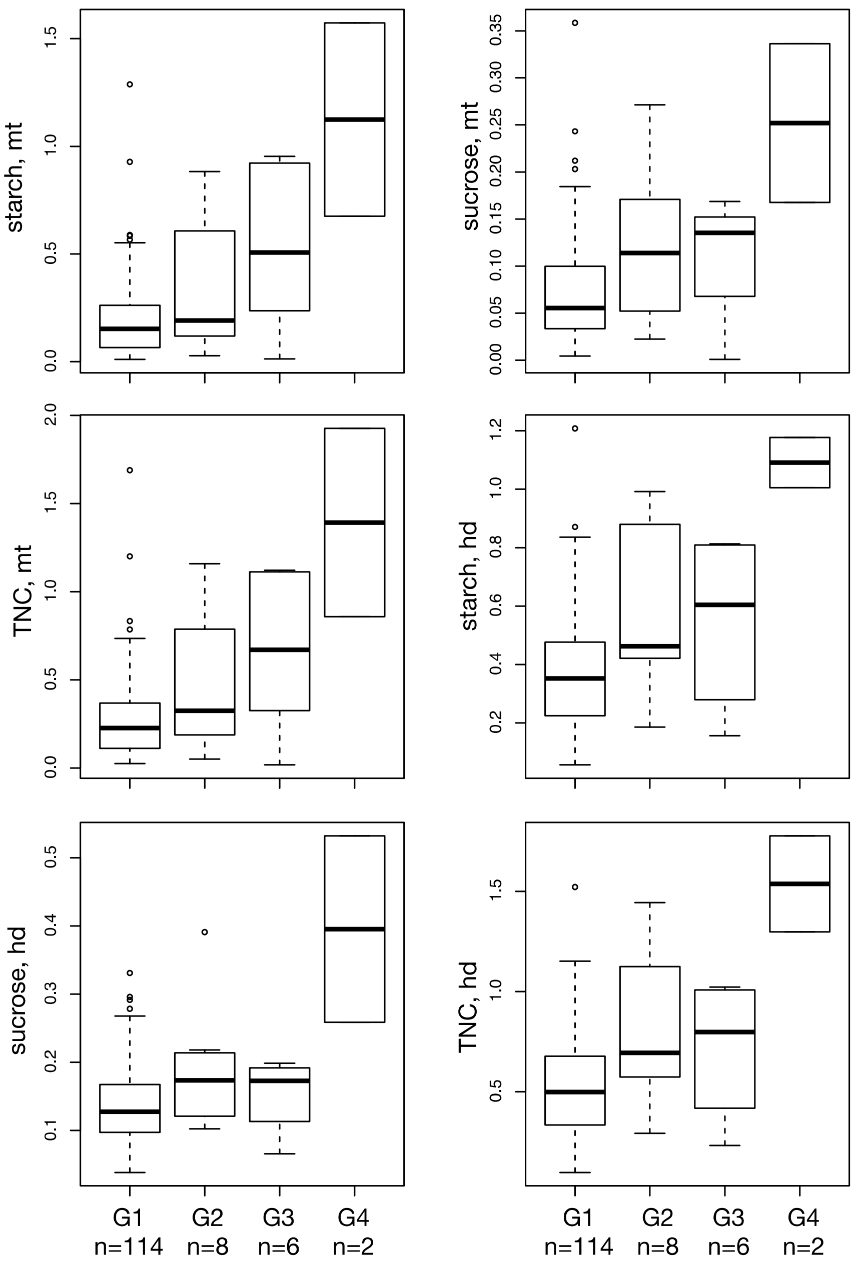

Fig. S14 Sequence polymorphisms cataloged in LOC_Os05g32710, isoamylase. Target region includes gene (chr 5: 19157600 - 19154970 bp) and 1.5kb upstream putative promoter (chr 5: 19159100 - 19157600 bp). A) All SNPs and indels found between cv. Curinga and *O. rufipogon* (IRGC105491), parents of the CSSL population used in this study. The blue annotation marks a putative repeat region, e.g. Simple Sequence Repeats (SSRs) annotated in OryzaRepeats and was called as a region of missing data in Curinga versus *O. rufipogon* at chr 5: 19157946-19157965 bp (potentially a 20bp indel). All polymorphisms are annotated as a *O. rufipogon* to Curinga change. The single SNP within the coding region results in an amino acid change of residue 726 from serine (*O. rufipogon*) → glycine (Curinga). B) Non-synonymous SNPs (nsSNPs) and indels within the same region (chr 5: 19159100 - 19157600 bp) across 484 re-sequenced *tropical japonica* accessions from the 3000 Rice Genomes Project. Here, the putative SSR region is denoted as a 22bp indel at the same position. All SNPs in this sub-figure are denoted as a change from the major allele to the minor allele in the population. Values within parentheses refer to the minor allele frequency. For synonymous SNPs in the coding region and SNPs in the putative promoter region, please refer to **Table S21**.

A All SNPs and indels between cv. Curinga and *O. rufipogon* (IRGC 105491):

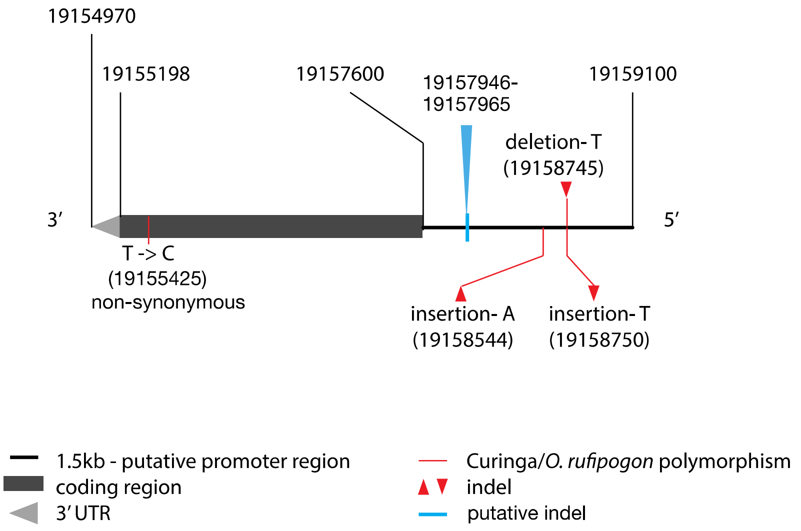

B nsSNPs and indels among 484 diverse *tropical japonica* accessions:

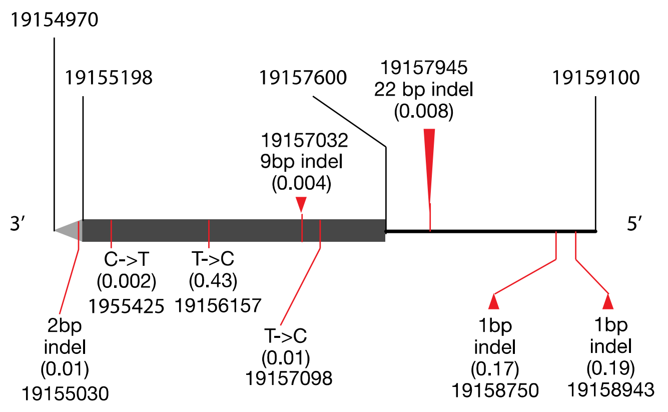

Fig. S15 Distribution of chromosome 5 msSNP alleles across U.S rice. Distribution of alleles for msSNP S5_19425787 across U.S. rice (red = rare 'A' allele in US-TRJ; orange= rare 'A' allele in US Indica varie)es; blue = common 'G' allele; gray = un-genotyped). Pedigrees begin approximately in the early 1900s and ends at present day.

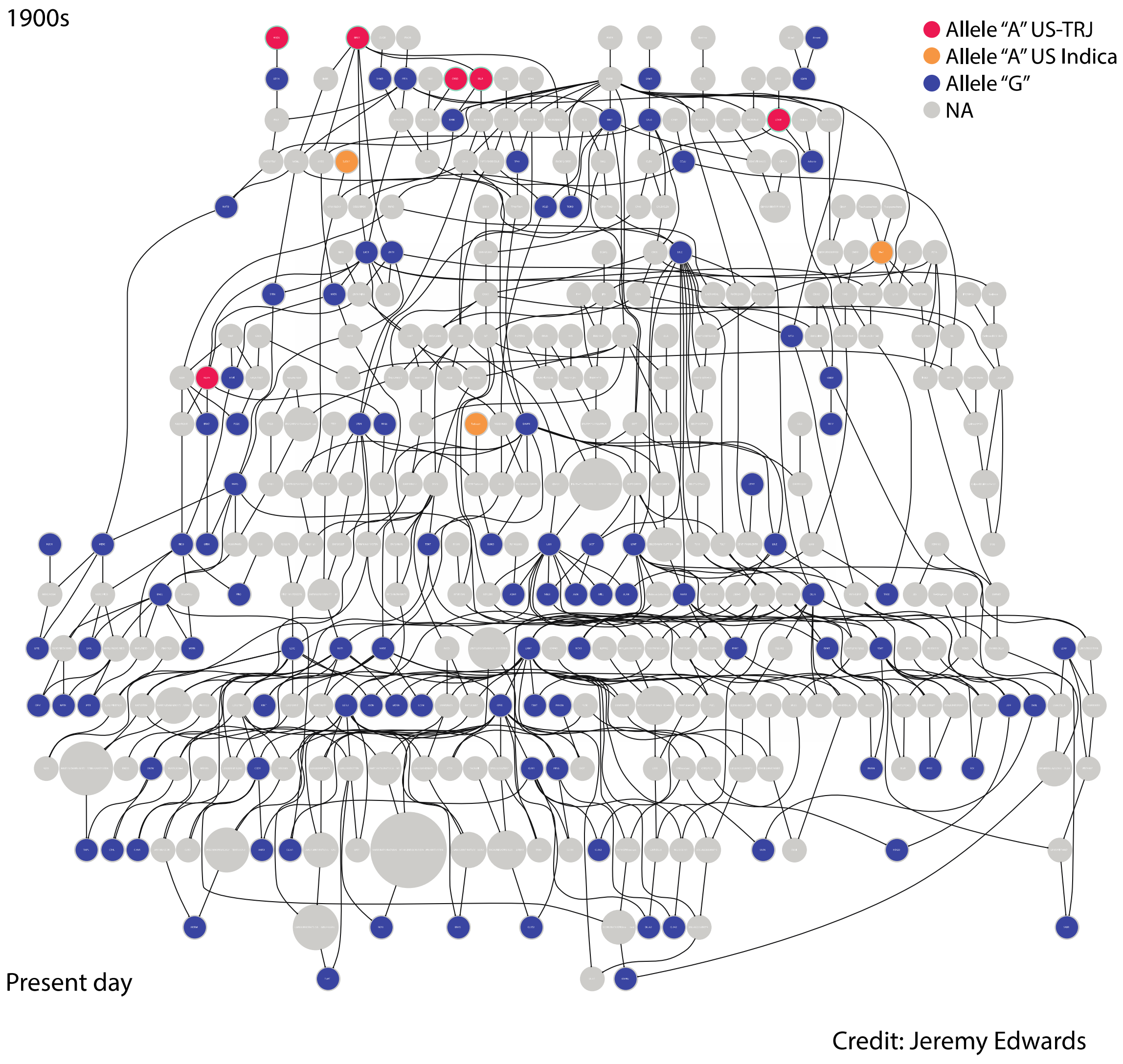

Fig. S16 Haplotype analysis of chromosome five QTL associated with sucrose-at-maturity in US-TRJ. Six accessions harbored the minor allele at msSNP, S5_19425787, that was associated with increased sucrose-at-maturity levels. All six accessions were varieties of the *tropical japonica-1* subpopulation according to FastStructure analysis. Top: Markers from 18-20Mb were extracted for these varieties along with 27 *indica* accessions and 30 *trj*-1 accessions. A consensus sequence was determined for *indica* and *trj*-1 and filtered to keep only markers that distinguished the two sequences. Next, similarity (defined as number of shared alleles/total markers) of each of these accessions with the trj-1 consensus sequence was calculated using windows of 10 SNPs, with step size 2 variants. Carolina Gold had missing data and was omitted for this analysis. Bottom: haplotypes of these six accessions.

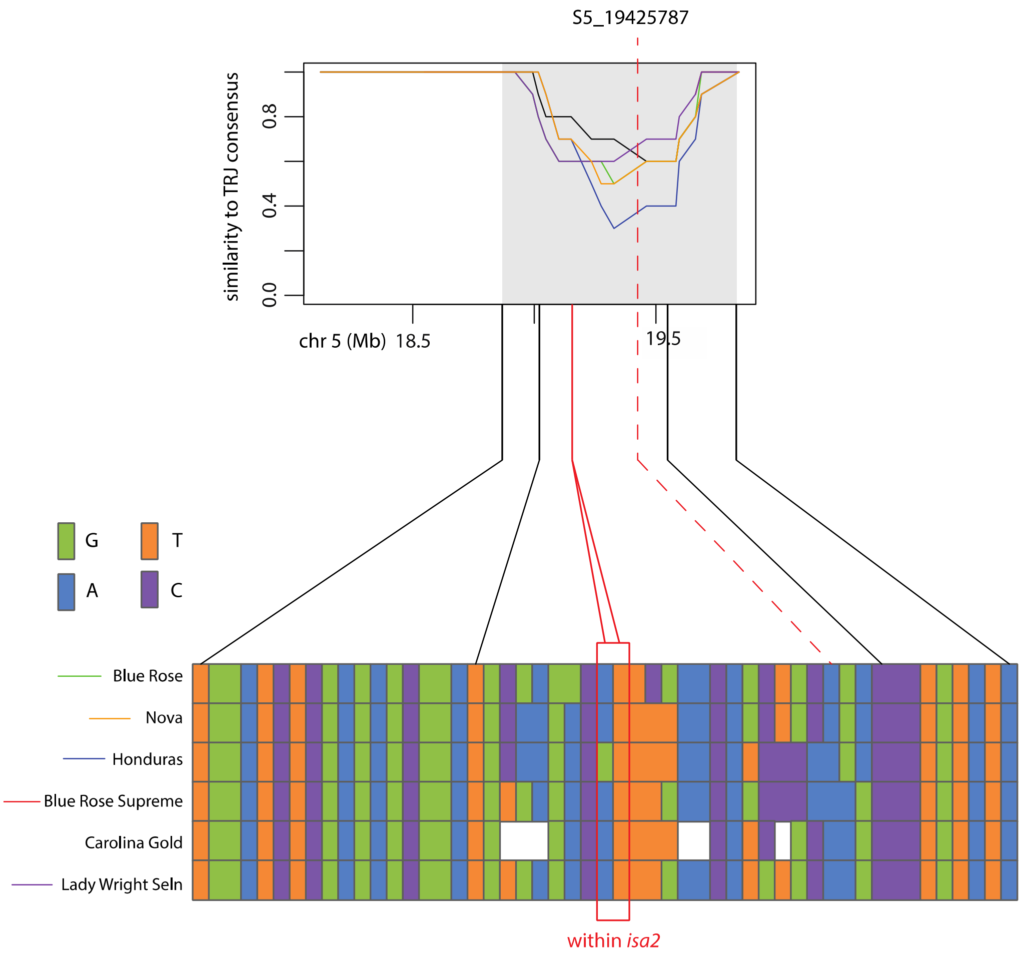

Supporting Information Tables S1-S121 may be found in the excel file 'Tables_S1-S21.xlsx' in separate worksheets.

Supporting Information Methods S1 may be found in the excel file 'Methods_S1.xlsx'

Supporting Information Methods S2 may be found as a zip file ('Methods_S2.zip') containing scripts and R binary files that can be used to replicate the NIR prediction analysis.
