# Supplementary material for: The buffering capacity of stems: genetic architecture of nonstructural carbohydrates in cultivated Asian rice, Oryza sativa
Source: New Phytol. 2017 May 30;215(2):658–71. doi: 10.1111/nph.14614 (PMC5488208; doi:10.1111/nph.14614)
Supplement: Supplementary file 4 — Methods S2 Zip file that contains full description of NIR prediction analysis, spectral data file, and relevant code. [file NPH-215-658-s004.zip › Methods_S1/NSC_Prediction_Pipeline.pdf]

Rice stem NSC prediction using NIR spectra

Edward J. Wolfrum and Peter DeWitt

November 14, 2016

```
##                _
## platform       x86_64-apple-darwin13.4.0
## version.string R version 3.2.4 (2016-03-10)
```

Description

This document details the analyses used to predict non-structural carbohydrate (NSC) traits in rice stem samples using near-infrared (NIR) spectral data. It utilizes publically available R packages and custom scripts that are included along with this supplemental information file. The calibration models used for prediction were published previously (Wang et al. 2016; doi: 10.1093/jxb/erw375).

Note: Functions utilized here are continuously being improved upon (i.e. consider them as 'beta' code).

1. Load R packages and helper functions

```
library(pls)
library(xlsx)
library(prospectr)
library(signal)
source("pca_Mar14-2.R")
source("Outlier_V3.R")
source("pls_uncertainties.R")
```

2. Import spectral data of prediction set

Load dataframe called 'theASRSData'. There are 975 observations and 2145 variables.

```
load("GWAS_Spectra.Rbin")
dim(theASRSData)
```

```
## [1]  976 2145
```

3. Data preparation

Define columns containing spectral wavelengths of interest for using in the prediction model. Spectral data are limited to the 4000-9000 wave-number range.

```
w1                              <- which(colnames(theASRSData)==4000)
w2                              <- which(colnames(theASRSData)==9002)
aNIRp                           <- theASRSData[,w1:w2]
theMeta                         <- theASRSData[,1:10]
```

Subject data to mathematical pre-treatment using the standard-normal-variate (SNV) scatter correction and a first-derivative Savitzky-Golay smoothing (n=25 points).

```
aNIR_SNV_DT_SG      <-  savitzkyGolay(detrend(aNIRp,as.numeric(colnames(aNIRp))),m=1,p=2,w=25)
```

Build dataframe for prediction.

```
theNIR              <-data.frame(1:dim(aNIRp)[1])
theNIR$spec2        <-as.matrix(aNIR_SNV_DT_SG)
theNIR              <-theNIR[,-1]
```

4. Load calibrated models

Previously calibrated models trained using 300 rice stem samples and externally validated with 134 rice stem samples (Wang et al. 2016, doi: 10.1093/jxb/erw375). Use **theModel.2** for PLS2 and **theModel2.2** for PLS1 model.

```
load("NSC_NIR_models.Rbin")
```

5. PLS2 Model: Multivariate prediction of stem starch and sucrose

The PLS2 Model predicts both starch and sucrose relative amounts (w/w) using 7 PCs. We first make predictions (values and scores in model space) and then calculate uncertainty values associated with each prediction. Uncertainties are characterized using the empirical U-deviation method (Zhang and Garcia-Munoz, 2009), which calculates multivariate confidence intervals (CIs).

```
theModel= theModel.2 ;   thePCs=7;    theName= "NSC_PLS2_model"
thePreds            <- predict(theModel,newdata=theNIR, ncomp=thePCs, type="response")
thePreds.scores     <- predict(theModel,newdata=theNIR, ncomp=thePCs, comps=1:thePCs, type="scores")
theU                <- PLSUncertainties(theModel, newdata=theNIR, ncomp=thePCs)
```

Plot distribution of predictions:

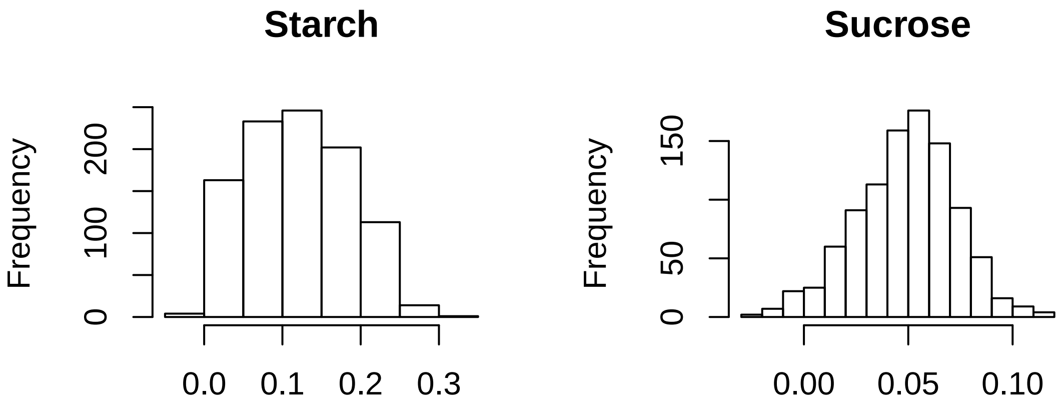

Calculate uncertainty measures relative to RMSEC.

```
theRMSEC        <-RMSEP(theModel,estimate="train",ncomp=thePCs)$val[,,2]
UU2             <-apply(theU$uncertainties$U.Deviation, 1, function(x) x/theRMSEC)
```

Plot the prediction uncertainties. We observe no values over 3.

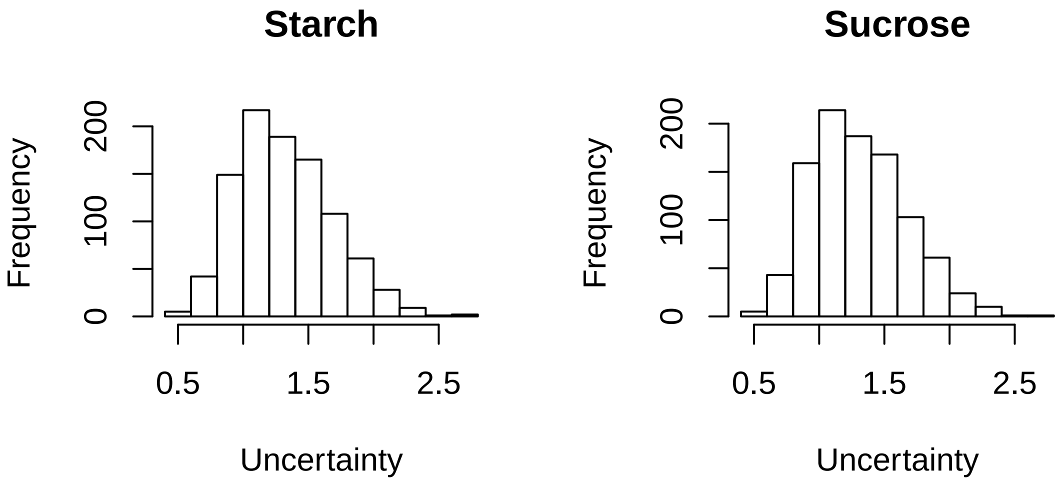

We extract all samples with uncertainty values over 2 to re-pack and re-scan as a conservative measure. We find 38 samples for sucrose and 42 for starch. Overall, there are 42 unique samples.

```
UU2<-data.frame(t(UU2))
lim=2
suc.outliers= which(UU2[,2]> lim)
starch.outliers= which(UU2[,1]> lim)
all.outliers= unique(c(suc.outliers, starch.outliers))
length(all.outliers)
```

```
## [1] 40
```

6. PLS1 Model: Univariate prediction of stem total non-structural carbohydrates (TNC)

The PLS1 Model predicts TNC, the sum of starch, sucrose, and glucose. As for the PLS2 model, we first make predictions (values and scores in model space) and then calculate uncertainty values associated with each prediction.

```
theModel = theModel2.2 ;    thePCs  = 5;      theName     = "TNC_PLS1_model"
thePreds              <- predict(theModel,newdata=theNIR, ncomp=thePCs, type="response")
thePreds.scores       <- predict(theModel,newdata=theNIR, ncomp=thePCs, comps=1:thePCs, type="scores")
theU                  <- PLSUncertainties(theModel, newdata=theNIR, ncomp=thePCs)
```

Plot distribution of predictions:

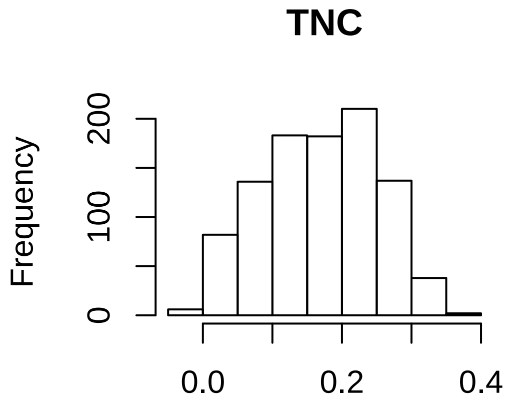

Calculate uncertainty measures relative to RMSEC.

```
theRMSEC         <-RMSEP(theModel,estimate="train",ncomp=thePCs)$val[,,2]
UU2              <-apply(theU$uncertainties$U.Deviation, 1, function(x) x/theRMSEC)
```

Plot the prediction uncertainties. We observe no values over 3.

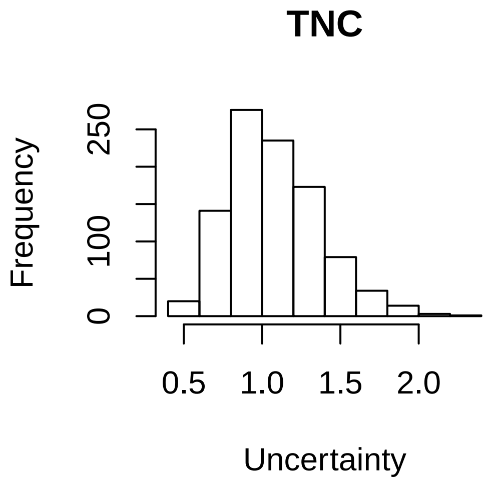

We extract all samples with uncertainty values over 2. There are 5 samples.

```
lim=2
tnc.outliers= which(UU2> lim)
length(tnc.outliers)
```

```
## [1] 4
```

7. Outlier samples: re-analysis

Overall, we found that we had very good predictions with low uncertainty values (all <3). However, to err on the conservative side, we elected to re-pack and re-scan samples that had uncertainties >2. There were 43 unique samples that had uncertainties >2 for sucrose and/or starch and/or TNC.

We predict these 43 re-scanned samples using the same PLS2 and PLS1 models as above. First we check the PLS2 (starch and sucrose) model.

We find that 20 of the 43 samples now have uncertainties <2. The rest are >2. Now we check PLS1 model for TNC.

Only a single sample has an uncertainty value >2 for TNC. We now plot original vs. new predictions.

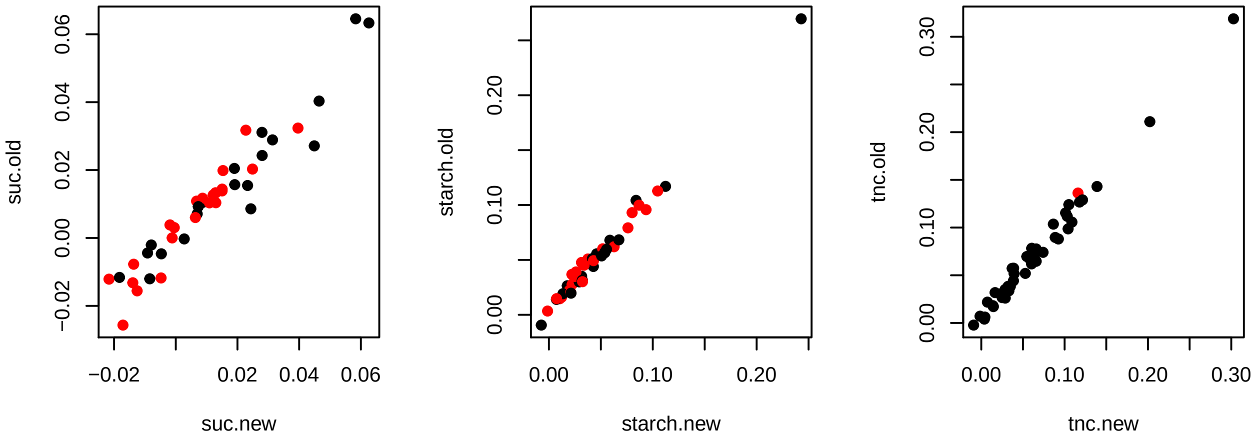

Red samples mark the re-scans that still had uncertainty values >2. TNC prediction uncertainties were greatly improved from re-packing and re-scanning. Original and new predictions were overall similar, despite some improvement in uncertainty. The least congruency was observed in sucrose, which is also the constituent that had the least predictive model during calibration. We went ahead and used these predictions for subsequent GWAS analysis; since these are not systematic prediction biases they should not increase chance of false positives in GWAS results.

Final note: functions used in estimating prediction uncertainties were updated on 10/30/2016 after GWAS took place. The functions provided with this supplemental file (pls_uncertainties) are the latest versions and thus give rise to slightly different values of uncertainties than original calculations, which were done prior to the update.
